# Burden of All Cancers Along With Attributable Risk Factors in China From 1990 to 2019: Comparison With Japan, European Union, and USA

**DOI:** 10.3389/fpubh.2022.862165

**Published:** 2022-05-26

**Authors:** Xiaorong Yang, Hui Chen, Shaowei Sang, Hao Chen, Lanbo Li, Xiaoyun Yang

**Affiliations:** ^1^Clinical Epidemiology Unit, Qilu Hospital of Shandong University, Jinan, China; ^2^Clinical Research Center of Shandong University, Qilu Hospital, Cheeloo College of Medicine, Shandong University, Jinan, China; ^3^Laboratory of Translational Gastroenterology, Department of Gastroenterology, Qilu Hospital of Shandong University, Jinan, China; ^4^Animal Laboratory, Qilu Hospital of Shandong University, Jinan, China

**Keywords:** cancer burden, disability-adjusted life years (DALYs), risk factors, temporal trend, epidemiology

## Abstract

**Background:**

Understanding the epidemiological characteristics of various cancers can optimize the prevention and control strategies in the national cancer control plan. This study aimed to report the burden differences, pattern trend, and potential risk factors of all neoplasm types in China in recent 30 years, and further compared with top economies in the world.

**Methods:**

The disability-adjusted life-years (DALYs) and age-standardized DALY rate (ASDR) of all neoplasms with the attributable risk factors from 1990 to 2019 in China, Japan, European Union, USA, and the world were extracted from the Global Burden of Disease Study 2019. The temporal trend analysis was estimated using the joinpoint regression model.

**Results:**

In 2019, about 251.4 million DALYs worldwide were caused by all neoplasms, and nearly 26.9% (67.5 million DALYs) occurred in China with the ASDR in 2019 of 342.09/10 000, which was higher than European Union (334.25/10 000), USA (322.94/10 000), and Japan (250.36/10 000). Although the cancer burden of the colorectum, non-Hodgkin lymphoma, oral cavity, ovary, and kidney in China was lower than in Japan, European Union and USA, the corresponding ASDR gradually increased in China over the past 30 years, but declined in the three developed areas. Around 46.29% of overall neoplasms DALYs in China in 2019 were attributed to 22 identified risk factors, and the specific risk attributable-fraction for several neoplasm types varied greatly in these regions.

**Conclusion:**

The ASDR of cancers of the lung, colorectum, pancreas, non-Hodgkin lymphoma, oral cavity, ovary, kidney, and chronic lymphoid leukemia increased in China compared to 30 years ago. With the population aging and the social transformation in China, the increasing burden of neoplasms and the changing spectrum of neoplasms suggest that effective comprehensive prevention and treatment measures should be adopted to reduce the burden, including public health education, strict tobacco-control policy, healthier lifestyles, along with expanding vaccination programs and early cancer screening.

## Introduction

Neoplasms have always been the main cause of the disease burden worldwide, which are responsible for about 10% of the total burden of all diseases in 2019 (1). Moreover, disability-adjusted life years (DALYs) caused by overall neoplasms continue to increase steadily from over the past decades, and is estimated to grow for the next 2 decades (1–3). Faced with the still severe burden of tumors, determining the scope of the problem and developing targeted solutions are the best way to reduce the burden of cancer in each national cancer control plan (4, 5). Some effective cancer prevention and treatment measures already exist, such as hepatitis B virus and human papillomavirus vaccines to prevent liver and cervical cancer (6, 7), or tyrosine kinase inhibitors for cancers with targeted mutations (8). However, these measures are usually very specific. Many factors, including demographic, cultural, environmental, behavioral and ecological exposure, as well as genetic susceptibility and tumor mutations, affect the temporal patterns of various cancers (2, 9). Therefore, a comprehensive understanding of the local cancer burden and related risk factors can optimize the formulation of the national cancer control plan, including governmental policies, resource allocation, research priorities and health system planning (10).

Recently, the International Agency for Research on Cancer (IARC) released the 2020 global cancer burden based on GLOBOCAN, with an estimated 19.3 million new cancer cases and 10.0 million cancer deaths (11). The current cancer situation in China remain an important public health focus due to its enormous sheer burden. In 2020, there were about 4.57 million new cancer cases and 3.00 million cancer deaths in China (12), which undoubtedly have a serious impact on the body function, mental health, financial ability and quality of life of many cancer patients and their families. The underlying reasons for the high cancer burden in China are as follows: First, China has the largest population in the world, estimated to be close to 1.45 billion, and the population is still rapidly aging (13). Second, with the urbanization and social-economic development over the past decades, the cancer spectrum shift is the most prominent in China (9). While the number of cancers continues to increase, the types of common cancers are also constantly changing, which increases the difficulty of cancer control, because the prevention and control strategies of different cancer types are very different (14). Third, due to local special living habits, some areas of China are more susceptible to certain types of cancer, which means that improving these cancer risk factors may help reduce the local cancer burden. For instance, people in southern China have a high risk of nasopharyngeal cancer, which may be related to eating large amounts of pickled fish and EBV infection (15). The age-standardized mortality rate of overall cancers in developed countries dropped gradually through decades of various efforts in cancer prevention and treatment (16). However, some common cancer types in developed countries, such as colorectal cancer, prostate cancer and breast cancer, have become common in China (17–20).

The Global Burden of Diseases (GBD) Study 2019 was performed by hundreds of professional collaborators to collect global disease burden data from original research studies and various databases, and subsequently provide systematical estimations on 369 diseases from 1990 to 2019 in 204 countries and territories (1, 21). In this study, the results on cancer burden from the GBD Study 2019 were used to analyze the difference of burden, pattern, trend, and potential risk factors of 36 neoplasm types from 1990 to 2019 in China. With the rapid economic development and the modernization of lifestyles, the transition in the cancer spectrum from developing countries to developed countries has become a major pattern in China (14). Comparison with top economies in the world, namely Japan, European Union, and USA can provide us with a more comprehensive picture in cancer burden. Understanding the tremendous achievements of developed countries in cancer prevention and treatment will help track the benefits of the national cancer control plan in China and optimize scientific programs to reduce the burden of tumors.

## Materials and Methods

### Data Source

We collected data on DALYs and the age-standardized DALY rate (ASDR) of all neoplasms in the global, China, Japan, European Union, and USA from 1990 to 2019 from the GBD Study 2019 *via* the Global Health Data Exchange query tool (https://ghdx.healthdata.org/gbd-results-tool). An exhaustive introduction of the original data sources and processing methods of the GBD 2019 study could be found in previous studies (1, 4). Each step used in the current study to analyze the GBD database complies with the Guidelines for Accurate and Transparent Health Estimates Reporting (GATHER) statements (Supplementary Material) (22). Briefly, the original cancer burden information by location and year in GBD study were collected from the single cancer registry system and aggregate database of cancer registries, including Cancer Incidence in Five Continents (CI5), Surveillance, Epidemiology, and End Results (SEER), and Nordic Cancer Registries database (NORDCAN). The China Disease Surveillance Points system and vital registration data collected by the Chinese Center for Disease Control and Prevention were the main original data sources for cancer estimation in China. All ICD9 and ICD10 codes identifying neoplasms (ICD9: 140-239 and ICD10: C00-D49, respectively) were included in the neoplasms estimation of the GBD study. According to the ICD codes, the neoplasms were categorized into 30 groups, and leukemia was further subdivided into five groups (Supplementary Table 1). The processing codes for estimating the cancer burden in the GBD study could be retrieved from the supporting website (http://ghdx.healthdata.org/gbd-2019/code/cod-2). The 95% uncertainty intervals (UIs) for all estimates were calculated based on the 25th and 975th ordered values of 1,000 random draws of the posterior distribution in the GBD Study. In order to comprehensively describe the regional disparity in neoplasm burden in provinces of China, data analysis was conducted on all 34 province-level subnational units, covering 22 provinces, five autonomous regions, four municipalities, Hong Kong Special Administrative Region (SAR), Macao SAR, and Taiwan. The provincial DALYs and age-standardized DALY rates of all neoplasms in the 34 provinces in China in 2017 were collected from a previous study (23).

### Risk Factor Analysis

The 69 specific risk factors at Level 4 in the GBD study were categorized into four Level 1 groups: environmental and occupational, behavioral, metabolic, and dietary risk factors (21). Among them, 34 risk factors were judged to have sufficient evidence to present the causal relationship with neoplasms development, and 13 occupational exposure risk factors were merged into the occupational carcinogens at Level 3 because of minor population attributable fractions. The attributable burden of potential risk factors in the GBD Study was estimated based on the comparative risk assessment framework (21). In short, the framework of comparative risk assessment contained six key steps: identifying cogent risk-outcome pairs, summarizing relative risk as a function of exposure, estimating the exposure levels and distributions, determining the theoretical minimum level of exposure, computing the population attributable fractions and attributable burden, and calculating attributable proportion for combined risk factors by considering the mediating effect.

### Statistical Analyses

To avoid the disparity of the age distribution of the populations, the ASDR was used to quantify the burden and trends of 36 neoplasms groups in different regions based on the GBD Standard Population Distribution (24). We performed joinpoint trend analysis to estimate the best-fit annual percentage changes (APC) in the ASDR of overall neoplasms from 1990 to 2019 in China, Japan, European Union, USA, and the world using the Joinpoint Regression Program (version 4.8.0.1). Joinpoint trend analysis fits a piecewise linear regression model to describe the adaptive changing trend with one or more line segments. In the final model, each segment informs a statistically significant change in trend (increase or decrease), which was tested *via* Monte Carlo permutation method. For understanding the overall changing trend of all cancer types, we further calculated the estimated annual percentage change (EAPC) to describe the overall temporal trend in ASDR of all cancer types based on the following regression model, ln (ASDR) = α + β^*^ calendar year + ϵ, and the EAPC with its 95% confidence interval (CI) were derived from the formula of 100 × (exp (β) – 1) (25). The main statistical analyses and graphing in this study were performed using R program version 4.0.3 (https://www.R-project.org/), and a two-sided *P*-value < 0.05 was considered statistically significant.

## Results

### Burden and Trend of Overall Neoplasms

An estimated 251.4 million DALYs worldwide in 2019 were attributed to all neoplasms, of which, nearly 26.9% (67.5 million DALYs) occurred in China (Table 1). The ASDR of all neoplasms in China (342.09/10 000) was somewhat close to the European Union (334.24/10 000) and USA (322.94/10 000), but far higher than that in the average world (306.24/10 000) and Japan (250.36/10 000). The overall neoplasms burden in Chinese men was relatively overwhelming, and the male DALYs in China (43.58 million) accounted for about 30.9% of the world (141.27 million DALYs; Supplementary Table 2). The male ASDR of all neoplasms in China (453.40/10 000) was more pronounced than that in the European Union (409.28/10 000), USA (368.28/10 000), worldwide (362.43/10 000), and Japan (315.84/10 000). In contrast, the female ASDR of overall neoplasms in China (240.97/10 000) was lower than that in the European Union (272.36/10 000), USA (285.72/10 000), and the global level (258.39/10 000), and only higher than Japan (196.01/10 000; Supplementary Table 3).

**Table 1:** The DALYs and age-standardized DALYs rate of all neoplasms in China, Japan, European Union, USA, and the world, 2019, both.

**Neoplasm type**	**China**		**Japan**		**European Union**		**USA**		**World**	
	**DALYs**	**ASDR**	**DALYs**	**ASDR**	**DALYs**	**ASDR**	**DALYs**	**ASDR**	**DALYs**	**ASDR**
	^*^10^5^ (%)	**/10^4^**	^*^10^5^ (%)	**/10^4^**	^*^10^5^ (%)	**/10^4^**	^*^10^5^ (%)	**/10^4^**	^*^10^5^ (%)	**/10^4^**
Neoplasms	675.2 (100)	342.09	74.17 (100)	250.36	310.0 (100)	334.24	166.5 (100)	322.94	2,514 (100)	306.24
Tracheal, bronchus, and lung cancer	171.3 (25.4)	83.13	13.47 (18.2)	40.80	67.47 (21.8)	71.25	41.86 (25.1)	76.74	458.6 (18.2)	55.16
Colon and rectum cancer	63.95 (9.47)	32.06	10.55 (14.2)	34.30	38.45 (12.4)	38.52	17.61 (10.6)	33.89	242.8 (9.66)	29.55
Stomach cancer	98.25 (14.6)	48.11	8.93 (12.04)	28.26	14.52 (4.68)	15.04	3.88 (2.33)	7.57	222.2 (8.84)	26.84
Breast cancer	29.57 (4.38)	14.42	3.98 (5.37)	17.30	25.86 (8.34)	29.12	14.03 (8.43)	28.41	206.25 (8.2)	24.76
Other malignant neoplasms	20.71 (3.07)	12.29	2.1 (2.83)	10.12	10.63 (3.43)	14.12	5.57 (3.34)	13.38	134.4 (5.35)	17.04
Liver cancer	53.25 (7.89)	26.43	5.57 (7.51)	17.52	8.8 (2.84)	9.33	5.51 (3.31)	10.72	125.3 (4.98)	15.11
Leukemia	23.09 (3.42)	16.38	2.01 (2.71)	9.75	10.69 (3.45)	13.44	6.69 (4.02)	14.54	116.6 (4.64)	15.05
Esophageal cancer	57.60 (8.53)	27.75	2.56 (3.45)	8.58	8.24 (2.66)	8.82	4.72 (2.83)	8.91	116.7 (4.64)	13.98
Pancreatic cancer	28.05 (4.15)	13.66	6.05 (8.16)	19.25	20.43 (6.59)	20.9	11.46 (6.88)	21.25	115.5 (4.59)	13.96
Brain and central nervous system cancer	20.53 (3.04)	12.62	0.89 (1.2)	5.44	10.38 (3.35)	14.89	5.65 (3.39)	13.39	86.6 (3.44)	10.90
Prostate cancer	10.03 (1.49)	5.21	2.45 (3.3)	6.06	17.37 (5.6)	15.21	9.27 (5.57)	16.0	86.45 (3.44)	10.79
Cervical cancer	16.22 (2.40)	7.91	0.97 (1.31)	4.60	4.36 (1.41)	5.47	2.25 (1.35)	5.09	89.55 (3.56)	10.72
Non-Hodgkin lymphoma	13.06 (1.93)	7.10	2.33 (3.14)	7.74	8.6 (2.77)	9.58	6.08 (3.65)	11.81	69.91 (2.78)	8.65
Lip and oral cavity cancer	5.76 (0.85)	2.83	0.77 (1.04)	2.77	4.53 (1.46)	5.2	1.81 (1.09)	3.50	55.07 (2.19)	6.61
Ovarian cancer	8.35 (1.24)	4.05	1.27 (1.71)	5.59	7.95 (2.56)	8.8	4.27 (2.56)	8.30	53.6 (2.13)	6.43
Bladder cancer	8.16 (1.21)	4.19	1.49 (2.01)	3.94	9.87 (3.18)	9.19	3.84 (2.31)	6.81	43.93 (1.75)	5.42
Other leukemia	12.58 (1.86)	8.55	0.53 (0.71)	2.06	2.34 (0.75)	2.66	1.57 (0.94)	3.24	39.38 (1.57)	5.06
Kidney cancer	6.43 (0.95)	3.43	1.3 (1.75)	4.35	8.34 (2.69)	8.89	4.34 (2.61)	8.40	40.53 (1.61)	4.96
Gallbladder and biliary tract cancer	7.64 (1.13)	3.77	2.95 (3.98)	8.27	3.82 (1.23)	3.75	1.03 (0.62)	1.91	36.21 (1.44)	4.40
Acute myeloid leukemia	2.89 (0.43)	2.04	1.02 (1.38)	4.71	4.41 (1.42)	5.60	3.02 (1.81)	6.50	30.6 (1.22)	3.90
Larynx cancer	4.98 (0.74)	2.39	0.23 (0.31)	0.72	3.36 (1.08)	3.71	1.18 (0.71)	2.21	32.62 (1.3)	3.88
Other pharynx cancer	1.45 (0.21)	0.69	0.53 (0.71)	1.91	3.42 (1.1)	4.02	0.82 (0.49)	1.59	32.35 (1.29)	3.84
Acute lymphoid leukemia	5.75 (0.85)	4.77	0.29 (0.39)	2.36	1.21 (0.39)	2.52	0.73 (0.44)	2.27	26.61 (1.06)	3.60
Multiple myeloma	3.47 (0.51)	1.71	0.87 (1.17)	2.64	5.19 (1.67)	5.08	3.5 (2.1)	6.39	24.97 (0.99)	3.03
Nasopharynx cancer	9.12 (1.35)	4.56	0.2 (0.27)	0.80	0.74 (0.24)	0.94	0.32 (0.19)	0.70	23.35 (0.93)	2.80
Uterine cancer	3.64 (0.54)	1.77	0.65 (0.88)	2.61	3.69 (1.19)	3.76	2.5 (1.5)	4.65	23.29 (0.93)	2.80
Malignant skin melanoma	1.5 (0.22)	0.80	0.16 (0.22)	0.69	4.65 (1.5)	5.74	3.09 (1.86)	6.48	17.08 (0.68)	2.08
Other neoplasms	1.8 (0.27)	0.97	0.88 (1.19)	2.78	2.88 (0.93)	2.65	1.8 (1.08)	3.26	12.17 (0.48)	1.53
Thyroid cancer	1.87 (0.28)	0.97	0.34 (0.46)	1.10	1.08 (0.35)	1.18	0.62 (0.37)	1.24	12.32 (0.49)	1.50
Non-melanoma skin cancer	3.24 (0.48)	1.68	0.2 (0.27)	0.58	1.16 (0.37)	1.10	1.49 (0.89)	2.68	11.83 (0.47)	1.47
Hodgkin lymphoma	0.86 (0.13)	0.50	0.11 (0.15)	0.46	0.88 (0.28)	1.35	0.49 (0.29)	1.23	11.46 (0.46)	1.44
Chronic myeloid leukemia	0.41 (0.06)	0.25	0.1 (0.13)	0.45	0.67 (0.22)	0.77	0.33 (0.2)	0.70	10.5 (0.42)	1.33
Chronic lymphoid leukemia	1.47 (0.22)	0.78	0.06 (0.08)	0.18	2.05 (0.66)	1.90	1.05 (0.63)	1.83	9.48 (0.38)	1.17
Mesothelioma	0.79 (0.12)	0.39	0.3 (0.4)	0.98	2 (0.65)	2.02	0.59 (0.35)	1.07	6.68 (0.27)	0.81
Testicular cancer	0.51 (0.08)	0.34	0.05 (0.07)	0.46	0.59 (0.19)	1.17	0.27 (0.16)	0.81	5.62 (0.22)	0.71

*DALYs, disability-adjusted life-years; ASDR, age-standardized DALY rate*.

**Figure 1:**
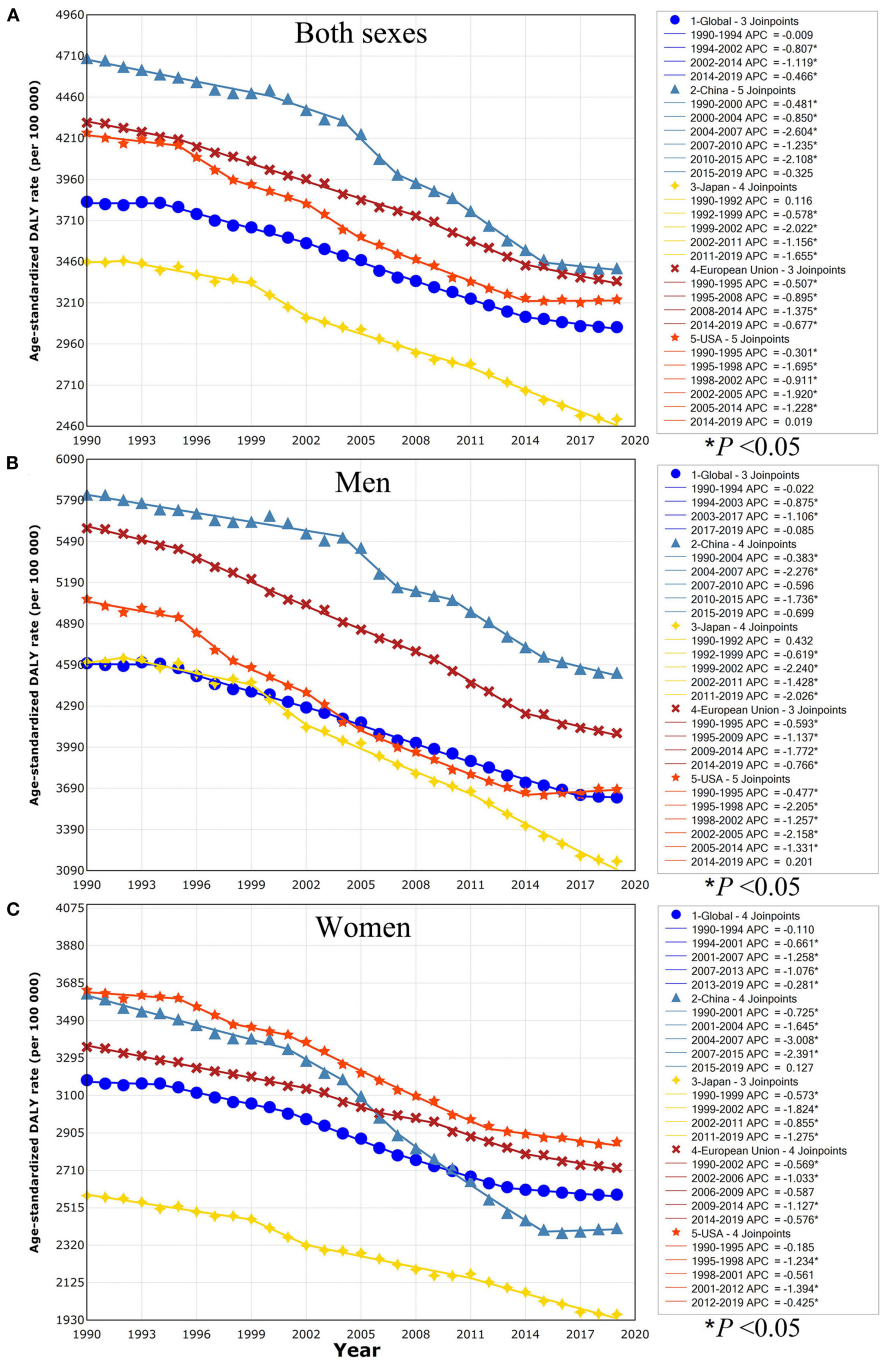
Trends for the ASDR of overall neoplasms in the world, China, Japan, European Union, and USA from 1990 to 2019 calculated by joinpoint regression analyses. **(A)** Both sexes; **(B)** men; **(C)** women. ASDR, age-standardized disability-adjusted life-year rate; APC, annual percentage change. ^*^P < 0.05 indicates a statistically significant change in trends (increase or decrease).

The changing trends of overall neoplasms obtained by the joinpoint regression analyses were shown in Figure 1. In China, the ASDR of overall neoplasms changed in six stages, and the former five stages presented an obvious downward trend, especially from 2004 to 2015. However, the corresponding ASDR kept stable with an APC of −0.325 (*P* > 0.05) in the final stage from 2015 to 2019 (Figure 1A). A similar pattern was detected in China stratified by gender, and also be found in the male population in USA with an APC of 0.201 (*P* > 0.05; Figures 1B,C). But the ASDR of overall neoplasms continued to decline in Japan, European Union, and the worldwide, regardless of men and women, and American women, over the past few decades.

### Tumor Types Composition

Regarding specific neoplasm types, these countries showed different patterns (Table 1, Supplementary Tables 2, 3). In 2019, for both sexes combined, the eight cancers with the heaviest burden in China were dominated by lung (25.4% of total DALYs), stomach (14.6%), colorectum (9.47%), esophageal (8.53%), liver (7.89%), breast (4.38%), pancreatic cancer (4.15%), and leukemia (3.42%). The proportion of DALYs for specific cancer in China exceeded 35% worldwide in 2019, including esophageal (49.4%), stomach (44.2%), liver cancer (42.5%), nasopharynx (39.1%), and lung cancer (37.4%) (Table 1), which were more pronounced in Chinese male patients (Supplementary Table 2). On the contrary, the Chinese proportion of DALYs for specific cancer in the world was <10% in 2019, including chronic myeloid leukemia (3.9%), other pharynx cancer (4.5%), Hodgkin lymphoma (7.5%), malignant skin melanoma (8.8%), testicular cancer (9.1%), and acute myeloid leukemia (9.4%; Table 1).

**Figure 2:**
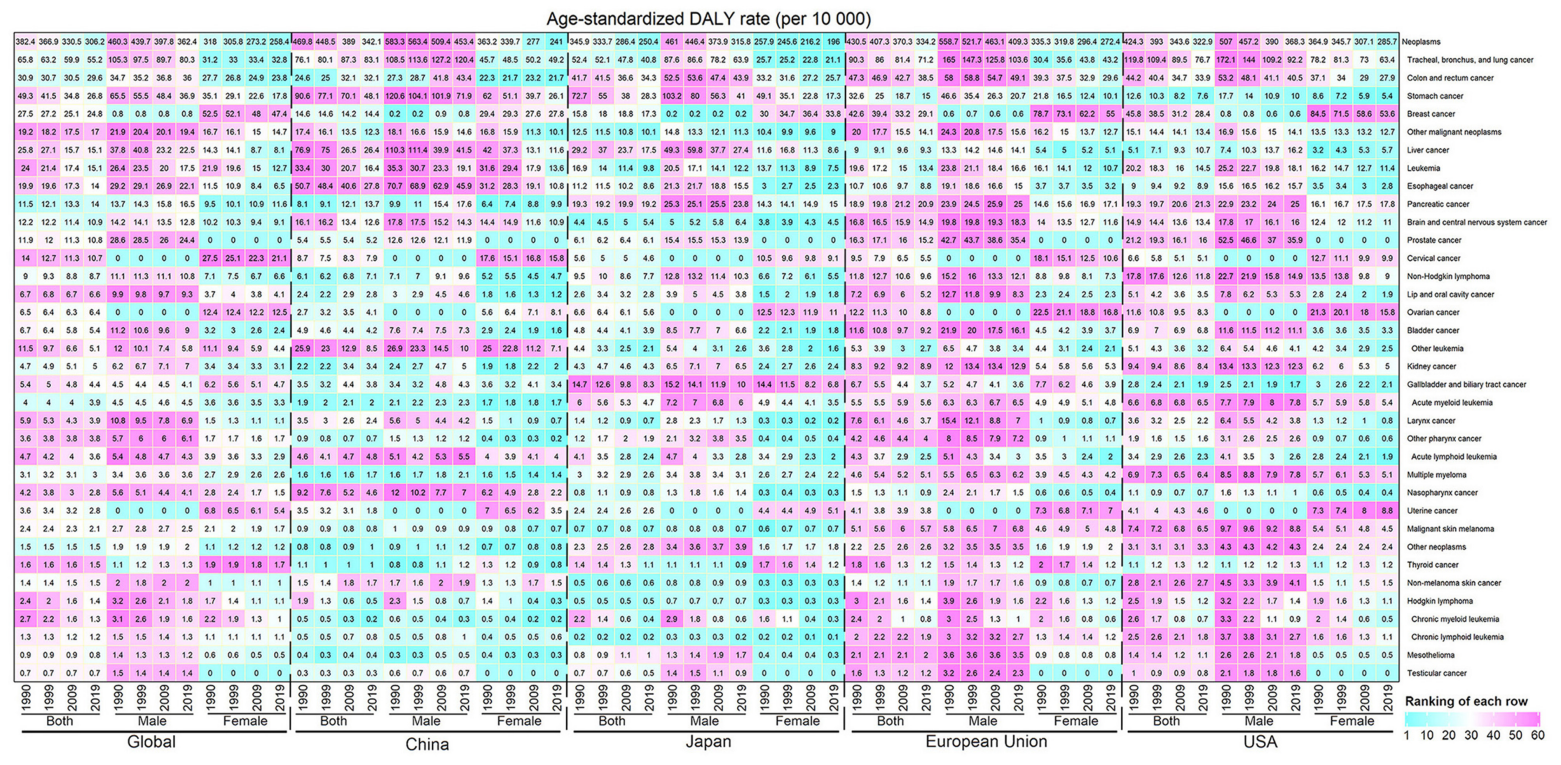
The ASDR distribution of 36 neoplasm types in the world, China, Japan, European Union, and USA were stratified by gender in 1990, 1999, 2009, and 2019. The color represents the ranking of each row (cyan is low and magenta is high).

Figure 2 showed the ASDRs of 36 neoplasms groups in the world, China, Japan, European Union and USA stratified by gender in 1990, 1999, 2009, and 2019. The burden patterns of most cancers in China were quite different from those in Japan, European Union, and USA. Compared with Japan, the burden distribution of most cancers in China presented an opposite relationship, except for oral cancer, bladder cancer, and malignant skin melanoma. For example, the cancer burden located in the lung, esophagus, liver, and nasopharynx in China was significantly higher than that in Japan, while the cancer burdens of the breast, pancreas, ovary, gallbladder, and biliary tract were heavier in Japan. The cancer patterns in European Union and USA were almost consistent. Except the cancer burden located in the stomach, esophagus, liver, cervical, nasopharynx, and other leukemia in China was more pronounced than European Union and USA, other cancers burden was heavier in European Union and USA.

**Figure 3:**
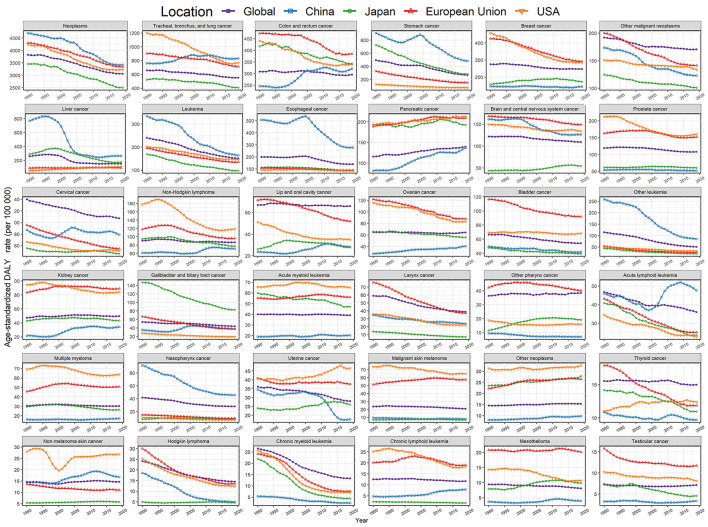
Trends for the ASDR of 36 neoplasm types in the world, China, Japan, European Union, and USA from 1990 to 2019, both.

Fortunately, the ASDR of most cancer types in China continued to decline over the past 30 years (Figure 3, Supplementary Figures 1, 2, Supplementary Tables 4–6). However, we noticed that the ASDR of lung cancer in China rose rapidly from 1990 to 2009, and exceeded the burden of disease in European Union and USA (Figure 3). Although the cancer burden of the colorectum, non-Hodgkin lymphoma, oral cavity, ovary, and kidney in China was lower than in Japan, European Union, and USA, the corresponding ASDR gradually increased in the past 30 years, on the contrary, it had been declining in these three developed areas. The ASDRs of most cancer types in Japan, European Union and USA significantly dropped in the past 30 years, but we observed an increase in the burden of pancreatic cancer in China, Japan, European Union and USA, and the ASDR of uterine cancer in Japan and USA also rose. In addition, the absolute DALYs of overall neoplasms and most specific neoplasm types incrementally rose in the four regions due to population aging and growth (Supplementary Figure 3).

### Neoplasms Burden in Subnational Areas in China

**Figure 4:**
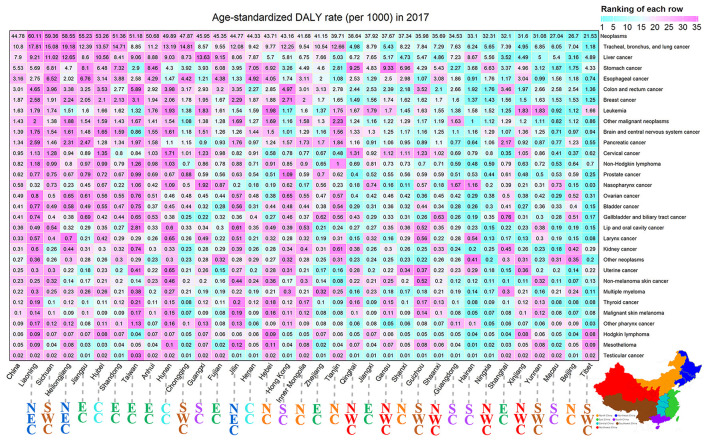
The ASDR distribution of 36 neoplasm types in China and its 34 provinces in 2017, both. The color represents the ranking of each row (cyan is low and magenta is high). NC, North China; NEC, Northeast China; EC, East China; SC, South China; CC, Central China; SWC, Southwest China; NWC, Northwest China.

The ASDR of all neoplasms in the 34 provinces of China in 2017 was found in Figure 4. The variety of ASDR of overall neoplasms was close to 2.79 times across the 34 provinces of China, and the four provinces with the greatest ASDR of overall neoplasms were Liaoning (60.11/1000), Sichuan (59.36/1000), Heilongjiang (58.55/1000), and Jiangsu (55.23/1000). In contrast, the three provinces with the lowest ASDR were Tibet (21.53/1000), Beijing (26.70/1000), and Macau (27.04/1000). In terms of most specific neoplasm types, more obvious heterogeneity was observed at the provincial level. For instance, the variety in ASDR of lung cancer was more than 15 times, with the highest rate observed in Liaoning (17.81/1000), and the lowest rate observed in Tibet (1.18/1000). High-burden nasopharyngeal cancer was more common in southern China, including Guangxi, Guangdong, Hainan, and so on.

### Main Risk Factors for Neoplasms

It was estimated that around 46.29% of overall neoplasms DALYs (54.02% for men and 32.87 % for women) in China in 2019 were attributed to all identified environmental and occupational, behavioral, metabolic, and dietary risk factors in GBD study estimation (Table 2). The specific risk attributable-fraction contributing to overall neoplasms differ largely in the world, China, Japan, European Union, and USA, which also presented obvious gender disparity (Table 2). Smoking was the leading cause of total neoplasms worldwide, and primarily responsible for about 22.12% of the worldwide neoplasms DALYs (36.28% for men and 8.63% for women) in 2019. Moreover, 40.99, 32.23, 33.98, and 31.00% of male neoplasms were attributed to smoking in China, Japan, European Union, and USA, respectively, while this corresponding value in females was 6.90, 9.95, 19.08, and 23.32%, respectively.

**Table 2:** The proportion (%) of overall neoplasms DALYs attributable to risk factors in the world, China, Japan, European Union, and USA, 2019, stratified by sex.

**GBD risk factors**	**Global**			**China**			**Japan**			**European Union**			**USA**		
	**Both**	**Men**	**Women**	**Both**	**Men**	**Women**	**Both**	**Men**	**Women**	**Both**	**Men**	**Women**	**Both**	**Men**	**Women**
All risk factors	41.23	47.22	33.55	46.29	54.02	32.87	40.15	47.14	30.09	46.34	50.78	40.20	46.26	47.89	44.20
Smoking	22.12	32.68	8.63	28.56	40.99	6.90	23.07	32.23	9.95	27.62	33.98	19.08	27.46	31.00	23.32
Alcohol use	5.07	7.17	2.26	5.22	7.32	1.35	5.55	6.34	4.30	7.23	8.80	5.00	5.24	6.26	4.00
High body-mass index	4.37	4.16	4.56	3.74	3.71	3.70	2.77	2.90	2.50	5.54	5.41	5.56	7.19	7.44	6.74
Unsafe sex	3.50	NA	8.15	2.32	NA	6.52	1.84	NA	4.64	1.64	NA	3.90	1.58	NA	3.46
High fasting plasma glucose	3.41	3.32	3.52	2.78	2.77	2.86	2.80	3.16	2.31	4.63	4.64	4.62	6.24	6.18	6.29
Occupational carcinogens	2.76	3.98	1.28	2.28	2.57	1.82	3.62	5.64	0.97	5.24	7.90	1.90	4.27	6.48	1.95
Ambient particulate matter pollution	2.75	3.49	1.79	5.46	6.06	4.42	1.52	1.89	1.01	1.94	2.33	1.40	0.95	1.00	0.88
Diet low in whole grains	1.51	1.58	1.42	1.48	1.53	1.40	2.06	2.12	1.94	1.95	2.04	1.82	1.63	1.72	1.50
Diet low in milk	1.51	1.56	1.42	1.78	1.82	1.71	2.31	2.35	2.21	1.25	1.30	1.18	0.91	0.95	0.86
Secondhand smoke	1.26	1.07	1.51	2.07	1.59	2.95	0.87	0.76	1.04	1.17	1.18	1.15	1.01	1.01	1.01
Diet low in calcium	1.25	1.35	1.11	1.42	1.51	1.28	1.79	1.83	1.68	0.60	0.73	0.44	0.48	0.55	0.39
Diet low in fruits	1.18	1.46	0.80	1.50	1.76	1.04	1.22	1.60	0.64	1.01	1.21	0.72	1.11	1.26	0.92
Household air pollution from solid fuels	0.76	0.87	0.60	1.20	1.16	1.25	0.00	0.00	0.00	0.08	0.09	0.07	0.00	0.00	0.00
Diet high in red meat	0.74	0.53	1.02	0.80	0.62	1.13	0.57	0.39	0.83	1.27	0.86	1.81	1.25	0.87	1.68
Residential radon	0.74	0.91	0.52	0.92	0.99	0.79	0.25	0.32	0.17	1.19	1.41	0.88	1.05	1.10	0.98
Diet high in sodium	0.68	0.83	0.50	1.24	1.41	0.94	0.92	1.09	0.68	0.31	0.38	0.22	0.16	0.20	0.11
Drug use	0.63	0.68	0.57	0.85	0.78	0.97	1.56	1.76	1.20	0.65	0.76	0.49	1.12	1.31	0.89
Chewing tobacco	0.59	0.60	0.55	0.07	0.10	0.02	0.05	0.07	0.03	0.02	0.02	0.01	0.18	0.32	0.02
Low physical activity	0.49	0.37	0.64	0.30	0.23	0.42	0.75	0.60	0.96	0.79	0.58	1.05	0.44	0.20	0.71
Diet high in processed meat	0.29	0.29	0.29	0.12	0.12	0.13	0.74	0.74	0.73	0.67	0.68	0.66	0.76	0.79	0.71
Diet low in fiber	0.18	0.18	0.18	0.10	0.10	0.10	0.28	0.26	0.29	0.21	0.22	0.19	0.20	0.19	0.20
Diet low in vegetables	0.16	0.20	0.12	0.03	0.04	0.02	0.06	0.08	0.02	0.13	0.19	0.06	0.15	0.23	0.05

*DALYs, disability-adjusted life-years*.

The following leading risk factors for total neoplasms burden worldwide were alcohol use, high body-mass index, unsafe sex, high fasting plasma glucose, occupational carcinogens, ambient particulate matter pollution, diet low in whole grains, diet low in milk, secondhand smoke, diet low in calcium and diet low in fruits, which were responsible for ~5.07, 4.37, 3.50, 3.41, 2.76, 2.75, 1.51, 1.51, 1.26, 1.25, and 1.18% of the worldwide neoplasms DALYs in 2019, respectively (Table 2). Compared with Japan, European Union, and USA, the proportions of overall neoplasms DALYs attributable to female alcohol use, high body-mass index, high fasting plasma glucose, male occupational carcinogens, diet low in whole grains, drug use, low physical activity, diet high in processed meat were relatively lower in China, on the contrary, the contribution of most other risk factors was greater in China. Besides, compared with 1990, we also found that the contribution from the high body-mass index, high fasting plasma glucose, diet low in whole grains, smoking in China, secondhand smoking in China, ambient particulate matter pollution in China, alcohol use in Japan, occupational carcinogens in China and Japan, and drug use in USA significantly increased (Supplementary Table 7).

**Figure 5:**
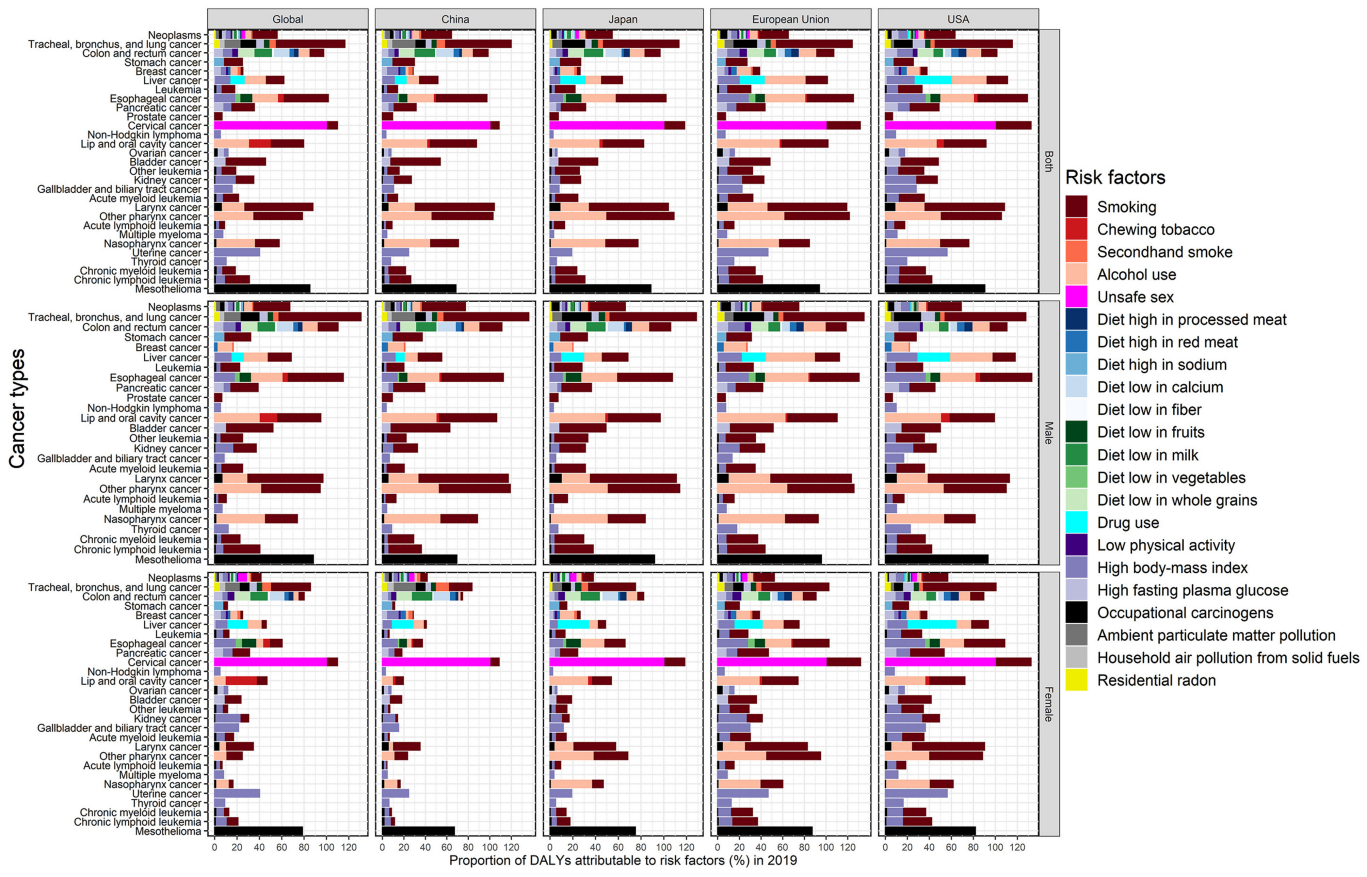
The population attributable fraction of all identified risk factors for 29 neoplasms groups in the world, China, Japan, European Union, and USA stratified by gender in 2019.

Figure 5 presented the attributable fraction of risk factors for 29 neoplasms groups in the world, China, Japan, European Union and USA stratified by gender in 2019. Smoking, alcohol drinking and high body-mass index played a pivotal role in the occurrence of many cancers across the world, such as lung, colorectal, esophageal, and pancreatic cancer, and so on, which presented greater contribution in women in European Union and USA than women in China and Japan. All cervical cancer burden was caused by unsafe sex and could be further aggravated by smoking with about 10% proportion. Further, ~85% of the global mesothelioma burden was attributable to occupational carcinogens, which was more pronounced in Japan, European Union, and USA.

## Discussion

The heterogeneous pattern in various neoplasm types in China leads to the complexity of tumor prevention and control (14, 26, 27). In the current study, we provided a comprehensive epidemiological description of the latest cancer burden, development trends, and risk factors in China from 1990 to 2019, and further comparisons with Japan, European Union, USA, and the world. Although the ASDR of overall neoplasms in China, Japan, the European Union, and USA presented a clear downward trend in the past three decades, the corresponding absolute DALYs due to neoplasms were still rising steadily, especially in China. Regarding specific neoplasm types, China was faced with a rising absolute burden in most cancer types except for Hodgkin lymphoma and chronic myeloid leukemia. Moreover, the ASDR of cancers of the lung, colorectum, pancreas, non-Hodgkin lymphoma, oral cavity, ovary, kidney, and chronic lymphoid leukemia increased from 30 years ago in China. Consequently, understanding the exact pattern of each cancer burden and its attributable risk facts is critical for the specific intervention measures and resource allocation in the national cancer control plan.

In general, the ASDR of most cancer types has continued to decline over the past 30 years in the four regions, especially in developed regions. This result might be explained by the following reasons. Firstly, the intervention of various risk factors effectively reduces the incidence of cancer. As reported by the GBD 2019 Risk Factors Collaborators, the disease burden attributable to most environmental and occupational risk factors was decreasing over the past decades, including smoking, unsafe sanitation, air pollution, malnutrition, dietary risks (21). For instance, smoking is a well-known cause of many diseases, including cancer, cardiovascular disease, and chronic respiratory diseases (28, 29). Facing the dangers of the tobacco epidemic to public health, the World Health Organization (WHO) promulgated the Framework Convention on Tobacco Control in 2003 to reduce tobacco consumption in the world, and the smoking prevalence has been steadily falling in most countries in recent years (28, 30). In addition, the population promotion of hepatitis B virus vaccine in China and human papillomavirus vaccine in developed countries have contributed to a marked reduction in the cancer burden in corresponding regions (31, 32). Secondly, the implementation of a population-based early cancer screening program can significantly reduce the subsequent burden of cancer, especially breast cancer, cervical cancer and alimentary canal cancers. Since the 1980's, the European Union and USA have successively implemented population-based screenings for breast cancer, cervical cancer, colorectal cancer, and so on across the country, which correspondingly led to the reduction of specific cancer burden (33, 34). Finally, the clinical applications for novel cancer treatments, including checkpoint inhibitors, tyrosine kinase inhibitors and chimeric antigen receptor (CAR) T cell, are associated with remarkable therapeutic response rates (35, 36). For example, before 2000, the treatment of chronic myeloid leukemia was limited to non-specific drugs, including busulfan, hydroxyurea and interferon-alpha (IFN-a), while the emergence of tyrosine kinase inhibitor reduced the corresponding annual mortality from 10–20% to 1–2% (37, 38).

On the contrary, the age-standardized cancer burden of the lung, colorectum, non-Hodgkin lymphoma, oral cavity, ovary, and kidney in China presented an overall upward trend in the past three decades, even partially surpassing Japan, European Union and USA. With social and economic development, urbanization, and changes in lifestyles and diets in China, these cancer patterns showed a significant shift from poverty-related cancers to wealth-related cancers, which could be reflected by the cancer disparity between urban and rural areas in China (39). Another noteworthy aspect is that higher cancer registration coverage and wider cancer diagnosis may contribute to the increase in some specific cancer burdens (40). In addition, inadequate control of potential risk factors can explain this phenomenon. For example, albeit many measures have been implemented to reduce the prevalence of smoking in China, the current male smoking prevalence in China was far higher than in Japan, European Union and USA, which partly explains the ASDR of lung cancer in China surpassed the three regions in recent years (25). Our result also indicated that the contribution from the high body-mass index, high fasting plasma glucose, diet low in whole grains, smoking, ambient particulate matter pollution, occupational carcinogens significantly increased in China over the past 30 years, which also partly explains the growing trend of lung and colorectal cancers. However, the detailed reasons need to be systematically evaluated in the future, and the surveillance of these cancers will need attention.

China is now shifting its focus from aspiring swift economic growth in the past to a coordinated economic development based on public health, which was declared in Healthy China 2030 blueprint (41). According to current trends and forecasts of cancer burden, China still has a long way to go before controlling the burden of cancer below the global average level, especially in the context of rapid population aging (42–44). The successful experience from developed countries will provide an important guideline for China's national cancer control plans. Among them, the intervention of potential risk factors and early cancer screening have achieved important profitable stories in the prevention and treatment of various cancer types, including cervical, colorectal, esophageal, and gastric cancer, etc. (2). Our results show that the overall cancer burden in Japan has been always significantly lower than in other economies, especially in lung cancer, leukemia, brain cancer, larynx cancer, etc. In addition, Japan has achieved great success in gastric cancer, thyroid cancer, and testicular cancer. The potential causes include the special geographical environment, low salt and high seafood traditional diet, national H. pylori control, and advanced cancer diagnosis and treatment (10, 45, 46). In our result, the development of many cancers was attributable to smoking, and the high prevalence of Chinese men underlines to deepen efforts in tobacco control for the Healthy China 2030 blueprint (47). Moreover, in China, high body mass index, high fasting blood glucose, low grain diets, environmental particulate pollution and occupational carcinogens have all contributed to the increased cancer burden, which implies that the corresponding public health education, promotion and intervention should also be included in the national cancer control plans (48–50). Although the burden of cancer caused by modifiable infectious pathogens has not been estimated in GBD 2019 Study, Martel and colleagues estimated that 13% of all cancer cases worldwide in 2018 were attributable to infectious pathogens, including Helicobacter pylori, human papillomavirus, and hepatitis B virus, hepatitis C virus, and Epstein-Barr virus, and about one-third of worldwide infection-related cancer cases occurred in China (6). The most effective strategy to prevent infectious cancers is to widely promote effective vaccines against these corresponding pathogens, albeit several major challenges remain, such as financial burden, efficiency and safety of vaccines, and lack of public awareness, especially in rural areas (14, 51). Zou et al. conducted a cost-effectiveness analysis on HPV vaccine indicate that combined screening and vaccination is more cost-effective, and a reduction in the domestic HPV vaccine price is necessary for cervical cancer prevention in China (52).

Although China has not carried out nationwide cancer screenings so far, several community-based cancer screenings supported by the government have been carried out in high-risk areas since 2005, covering gastric, esophageal, colorectal, liver, lung, nasopharyngeal, cervical, and female breast cancer (14, 53). In addition, spontaneous cancer screenings are becoming more popular among urban residents, even at their own expense. The main obstacles to cancer screening in China are low national population coverage and bad population compliance, which implies that improving the coverage of the target population and the sensitivity of screening methods is critical to the effectiveness of early cancer screening. Establishing a suitable hierarchical screening strategy will undoubtedly optimize the current cancer screening strategy, namely, quantitatively and initially screening out the high-risk population from the community population-based on easy-to-collect risk factors, then applying fast and accurate technologies such as liquid biopsy to identify candidate cancer patients, and finally performing the gold standard diagnostic examination for early cancer treatment (54–57). However, the over-diagnosis of tumors, such as thyroid cancer and breast cancer still needs attention (58, 59).

Some limitations need to be considered when interpreting the results of our research. First, due to the lack of necessary data, some cancer subtypes such as gastric cardia and non-cardia gastric cancer, esophageal squamous cell carcinoma and esophageal adenocarcinoma have not been further analyzed, but the corresponding epidemiological characteristics, trends, and regional clusters present obvious heterogeneity (60). In addition, the neoplasms burden attributable to other important risk factors, especially infectious pathogens, has not been studied for the limited estimation in the GBD database. Martel and colleagues summarized the global cancer burden attributable to infections, which somewhat compensates for current deficiencies and promotes prevention efforts (6). Finally, the heterogeneity of diagnostic methods and tumor registrations in different countries or even sub-regions may bias our results. However, each national cancer registry collects information on all cancers with a clear diagnosis every year, which alleviates current worries to a certain extent.

## Conclusion

Compared with Japan, European Union, and USA, the cancer burden in China is relatively heavier, especially for men. With the aging of the population and the transformation of Western lifestyles in China, the increasingly severe burden of cancer and the changing cancer spectrum indicate that effective comprehensive prevention and treatment measures should be adopted to reduce the burden of cancer in China. The national cancer control plan implemented by the government should be adjusted according to the current cancer burden patterns and the evidence-based cancer practices established by developed countries, and at the same time, taking into account the diversity of cancer types in different regions of China. More public health education and population interventions are urgently demanded to bridge the gap between advanced evidence-based knowledge of cancer prevention and severe situation of all identified risk factors, especially in underdeveloped areas, which have proven to be the most cost-effective in long-term cancer control, including tobacco control, alcohol cessation, and healthy lifestyles. In addition, expanded vaccination programs and early cancer screening are also recommended. After all, cancer should be controlled sooner rather than later.

## Data Availability Statement

Publicly available datasets were analyzed in this study. This data can be found at: all data could be extracted from in the online GBD repository, http://ghdx.healthdata.org/gbd-results-tool.

## Ethics Statement

The studies involving human participants were reviewed and approved by Institutional Review Boards of Qilu Hospital of Shandong University. Written informed consent for participation was not required for this study in accordance with the national legislation and the institutional requirements.

## Author Contributions

XiaorY and XiaoyY: conception and design. XiaorY, HuC, and LL: data collection. XiaorY and HuC: data analyses. XiaorY, SS, and HaC: results visualization. All authors: results interpretations, manuscript writing, and final approval of manuscript.

## Funding

This work was supported by the Special Foundation for Science and Technology Basic Research Program (2019FY101103), National Natural Science Foundation of China (82103912), China Postdoctoral Science Foundation (2021M700080), and Shandong Provincial Natural Science Foundation (ZR2020QH302). The funder of the study had no role in study design, data collection, data analysis, data interpretation, or writing of the report. The corresponding author had full access to all of the data and the final responsibility to submit for publication.

## Conflict of Interest

The authors declare that the research was conducted in the absence of any commercial or financial relationships that could be construed as a potential conflict of interest.

## Publisher's Note

All claims expressed in this article are solely those of the authors and do not necessarily represent those of their affiliated organizations, or those of the publisher, the editors and the reviewers. Any product that may be evaluated in this article, or claim that may be made by its manufacturer, is not guaranteed or endorsed by the publisher.

## Abbreviations

DALYs: disability-adjusted life-years
GBD: Global Burden of Diseases
ASDR: age-standardized DALY rate
UI: uncertainty interval
SAR: Special Administrative Region
APC: annual percentage changes
EAPC: estimated annual percentage change.
