# Supplementary material for: Burden of All Cancers Along With Attributable Risk Factors in China From 1990 to 2019: Comparison With Japan, European Union, and USA
Source: Front Public Health. 2022 May 26;10:862165. doi: 10.3389/fpubh.2022.862165 (PMC9178089; doi:10.3389/fpubh.2022.862165)
Supplement: Supplementary file 1 [file Data_Sheet_1.PDF]

Burden of all cancers along with attributable risk factors in China from 1990 to 2019: comparison with Japan, European Union, and USA

Running title: Comparing cancer burden in top four economies

1 supplementary report checklist, 3 supplementary figures and 7 supplementary table

List of supporting information

Supplementary report checklist. GATHER checklist of information that should be included in reports of global health estimates.

Table S1: List of International Classification of Diseases (ICD) codes mapped to the Global Burden of Disease cause list for neoplasms.

Table S2. The DALYs and age-standardized DALYs rate of all neoplasms in China, Japan, European Union, USA, and the world, 2019, men.

Table S3. The DALYs and age-standardized DALYs rate of all neoplasms in China, Japan, European Union, USA, and the world, 2019, women.

Table S4. The estimated annual percentage change with 95% confidence interval in age-standardized DALYs rate of all neoplasms in China, Japan, European Union, USA, and the world, from 1990 to 2019, both sexes.

Table S5. The estimated annual percentage change with 95% confidence interval in age-standardized DALYs rate of all neoplasms in China, Japan, European Union, USA, and the world, from 1990 to 2019, men.

Table S6. The estimated annual percentage change with 95% confidence interval in age-standardized DALYs rate of all neoplasms in China, Japan, European Union, USA, and the world, from 1990 to 2019, women.

Table S7. The proportion (%) of cancer DALYs attributable to risk factors in the world, China, Japan, European Union, and USA, 1990, stratified by sex.

Figure S1. Trends for the ASDR of 36 neoplasm types in the world, China, Japan, European Union, and USA from 1990 to 2019, men.

Figure S2. Trends for the ASDR of 36 neoplasm types in the world, China, Japan, European Union, and USA from 1990 to 2019, women.

Figure S3. Trends for the absolute DALYs of 36 neoplasm types in China, Japan, European Union, and USA from 1990 to 2019, both. DALYs, disability-adjusted life-years.

GATHER checklist of information that should be included in reports of global health estimates.

#	Checklist item	Section/paragraph/ interpretation
**Objectives and funding**		
1	Define the indicators, populations, and time periods for which estimates were made.	Methods / "Data Sources" section
2	List the funding sources for the work.	Funding section
**Data Inputs**		
For all data inputs from multiple sources that are synthesized as part of the study:		
3	Describe how the data were identified and how the data were accessed.	Methods / "Data Sources" section, the details have been published previously.
4	Specify the inclusion and exclusion criteria. Identify all ad-hoc exclusions.	Methods / "Data Sources" section, the details have been published previously.
5	Provide information on all included data sources and their main characteristics. For each data source used, report reference information or contact name/institution, population represented, data collection method, year(s) of data collection, sex and age range, diagnostic criteria or measurement method, and sample size, as relevant.	Available via online data source tools (http://ghdx.healthdata.org/gbd-2019/data-input-sources).
6	Identify and describe any categories of input data that have potentially important biases (e.g., based on characteristics listed in item 5).	Methods / "Data Sources" section, the details have been published previously.
For data inputs that contribute to the analysis but were not synthesized as part of the study:		
7	Describe and give sources for any other data inputs.	Available via online data source tools (http://ghdx.healthdata.org/gbd-2019/data-input-sources).
For all data inputs:		
8	Provide all data inputs in a file format from which data can be efficiently extracted (e.g., a spreadsheet as opposed to a PDF), including all relevant	Available via online data source tools (http://ghdx.healthdata.org/gbd-2019/data-input-sources); input

	meta-data listed in item 5. For any data inputs that cannot be shared due to ethical or legal reasons, such as third-party ownership, provide a contact name or the name of the institution that retains the right to the data.	data not available in tools will be made available upon request.
**Data analysis**		
9	Provide a conceptual overview of the data analysis method. A diagram may be helpful.	Flow diagrams of the overall methodological processes were available online (http://ghdx.healthdata.org/gbd-2019/code/cod-2)
10	Provide a detailed description of all steps of the analysis, including mathematical formulae. This description should cover, as relevant, data cleaning, data pre-processing, data adjustments and weighting of data sources, and mathematical or statistical model(s).	As mentioned in the Methods / "Risk factor analysis" and "Statistical Analysis" section, the details have been published previously.
11	Describe how candidate models were evaluated and how the final model(s) were selected.	As mentioned in the Methods section, the details have been published previously.
12	Provide the results of an evaluation of model performance, if done, as well as the results of any relevant sensitivity analysis.	As mentioned in the Methods section, the details have been published previously.
13	Describe methods for calculating uncertainty of the estimates. State which sources of uncertainty were, and were not, accounted for in the uncertainty analysis.	Methods / "Statistical Analysis" section
14	State how analytic or statistical source code used to generate estimates can be accessed.	Methods / "Statistical Analysis" section
**Results and Discussion**		
15	Provide published estimates in a file format from which data can be efficiently extracted.	Results, and online data tools (data visualization tools, and data query tools, http://ghdx.healthdata.org/gbd-2019)
16	Report a quantitative measure of the uncertainty of the estimates (e.g. uncertainty intervals).	Results, and online data tools (data visualization tools, and data query tools,

		http://ghdx.healthdata.org/gbd-2019)
17	Interpret results in light of existing evidence. If updating a previous set of estimates, describe the reasons for changes in estimates.	Discussion, paragraphs 1-5
18	Discuss limitations of the estimates. Include a discussion of any modelling assumptions or data limitations that affect interpretation of the estimates.	Discussion, paragraph 6

Table S1: List of International Classification of Diseases (ICD) codes mapped to the Global Burden of Disease cause list for neoplasms

Neoplasm type	ICD10	ICD9
Neoplasms	C00-C07, C08-C19.0, C20, C21-C21.8, C22-C22.4, C22.7-C23, C24-C26.1, C26.8-C26.9, C30-C30.1, C31-C33, C34-C34.92, C37-C37.0, C38-C39.9, C40-C41.4, C41.8-C41.9, C43-C45.2, C45.7, C45.9, C47-C4A, C50-C50.629, C50.8-C52, C53-C54.3, C54.8-C56.2, C56.9-C58.0, C60-C64.2, C64.9-C69.92, C70-C70.1, C70.9-C73, C74-C75.5, C75.8-C79.9, C80-C81.49, C81.7-C81.79, C81.9-C85.29, C85.7-C86.6, C88-C90.32, C91-C93.7, C93.9-C95.2, C95.7-C97.9, D00-D24.9, D26.0-D39.9, D4-D49.9, E34.0, K51.4-K51.419, K62.0-K62.3, K63.5, N60-N60.99, N84.0-N84.1, N87-N87.9, Z03.1, Z08-Z09.9, Z12-Z12.9, Z80-Z80.9, Z85-Z85.9, Z86.0-Z86.03	140-175.9, 177-217.8, 219-237.6, 237.70-237.72, 237.9-239.9, 569.0, 610-610.9, 622.1-622.2, 622.7, V07.39, V10-V11, V13.22-V13.24, V16-V16.9, V42.4, V42.81-V42.82, V59.2-V59.3, V72.32, V76-V76.9
Lip and oral cavity cancer	C00-C07, C08-C08.9, Z85.81-Z85.810	140-145.9, V76.42
Nasopharynx cancer	C11-C11.9	147-147.9
Other pharynx cancer	C09-C10.9, C12-C13.9	146-146.9, 148-148.9
Esophageal cancer	C15-C15.9, Z85.01	150-150.9
Stomach cancer	C16-C16.9, Z12.0, Z85.02-Z85.028	151-151.9, 209.23, V10.04
Colon and rectum cancer	C18-C19.0, C20, C21-C21.8, Z12.1-Z12.13, Z85.03-Z85.048, Z86.010	153-154.9, 209.1-209.17, V10.05-V10.06, V76.41, V76.5-V76.52
Liver cancer	C22-C22.4, C22.7-C22.9, Z85.05	155-155.9, V10.07
Gallbladder and biliary tract cancer	C23, C24-C24.9	156-156.9
Pancreatic cancer	C25-C25.9, Z85.07	157-157.9
Larynx cancer	C32-C32.9, Z85.21	161-161.9, V10.21

Tracheal, bronchus, and lung cancer	C33, C34-C34.92, Z12.2, Z80.1-Z80.2, Z85.1-Z85.20	162-162.9, 209.21, V10.1-V10.20, V16.1-V16.2, V16.4-V16.40
Malignant skin melanoma	C43-C43.9, Z85.82-Z85.828	172-172.9
Non-melanoma skin cancer	C44.01-C44.99	173-173.99
Breast cancer	C50-C50.629, C50.8-C50.929, Z12.3-Z12.39, Z80.3, Z85.3, Z86.000	174-175.9, V10.3, V16.3
Cervical cancer	C53-C53.9, Z12.4, Z85.41	180-180.9, V10.41, V72.32
Uterine cancer	C54-C54.3, C54.8-C54.9, Z85.42, Z86.001	182-182.9
Ovarian cancer	C56-C56.2, C56.9, Z80.41, Z85.43	183-183.0, 183.8-183.9, V10.43, V16.41
Prostate cancer	C61-C61.9, Z12.5, Z80.42, Z85.46	185-185.9, V10.46, V16.42, V76.44
Testicular cancer	C62-C62.92, Z80.43, Z85.47-Z85.48	186-186.9, V10.47-V10.48, V16.43
Kidney cancer	C64-C64.2, C64.9-C65.9, Z80.51, Z85.52-Z85.54	189-189.1, 189.5-189.6, 209.24
Bladder cancer	C67-C67.9, Z12.6-Z12.79, Z80.52, Z85.51	188-188.9, V10.51, V16.52, V76.3
Brain and central nervous system cancer	C70-C70.1, C70.9-C72.9, Z85.841-Z85.848, Z86.011	191-191.9
Thyroid cancer	C73, Z85.850	193-193.9
Mesothelioma	C45-C45.2, C45.7, C45.9	
Hodgkin lymphoma	C81-C81.49, C81.7-C81.79, C81.9-C81.99, Z85.71-Z85.72	201-201.98, V10.72
Non-Hodgkin lymphoma	C82-C85.29, C85.7-C86.6, C96-C96.9	200-200.9, 202-202.98
Multiple myeloma	C88-C90.32	203-203.9
Leukemia	C91-C93.7, C93.9-C95.2, C95.7-C95.92, Z80.6, Z85.6	204-208.92, V10.59-V10.69, V16.6
Acute lymphoid leukemia	C91.0-C91.02, C91.2-C91.32, C91.6-C91.62	204.0-204.02
Chronic lymphoid leukemia	C91.1-C91.12	204.1-204.12
Acute myeloid leukemia	C92.0-C92.02, C92.3-C92.62, C93.0-C93.02, C94.0-C94.02, C94.2-C94.22, C94.4-C94.5	205.0-205.02, 205.2-205.32, 206.0-206.02, 207.0-207.02, 207.2-207.82
Chronic myeloid leukemia	C92.1-C92.22	205.1-205.12, 207.1

Other leukemia	C91.2-C91.9, C92.2, C92.7-C92.9, C93.1-C93.9, C94.1, C94.3, C94.6-C95.9	204.2-204.9, 205.2, 205.8-205.9, 206.2-207, 207.2-208.9
Other malignant neoplasms	C17, C30-C31, C37, C38, C40- C41, C47-C49, C4A, C51-C52, C57-C58, C60, C63, C66, C68, C69, C74-C75	152-152.9, 158-158.9, 160-160.9, 163-164.9, 170-171.9, 181-181.9, 183.2-183.5, 184-184.9, 187-187.9, 189.2-189.4, 189.8-190.9, 192-192.9, 194-194.8, 209-209.03, 209.22, 209.25-209.27, 209.31-209.36
Other neoplasms	C75.90-C75.92, D00-D24.9, D26.0-D39.9, D4-D49.9, E34.0, K51.4-K51.419, K62.0-K62.3, K63.5, N60-N60.99, N84.0-N84.1, N87-N87.9	209.4-209.57, 209.61, 209.63-209.67, 210.0-217.8, 219-237.6, 237.70-237.72, 237.9-239.9, 569.0, 610-610.9, 622.1-622.2, 622.7

Table S2. The DALYs and age-standardized DALYs rate of all neoplasms in China, Japan, European Union, USA, and the world, 2019, men.

Neoplasm type	China		Japan		European Union		USA		Worldwide	
	DALYs $*10^5$ (%)	ASDR $/10^4$	DALYs $*10^5$ (%)	ASDR $/10^4$	DALYs $*10^5$ (%)	ASDR $/10^4$	DALYs $*10^5$ (%)	ASDR $/10^4$	DALYs $*10^5$ (%)	ASDR $/10^4$
Neoplasms	435.82(100)	453.40	44.47(100)	315.84	177.02(100)	409.28	89.54(100)	368.28	1412.7(100)	362.43
Tracheal, bronchus, and lung cancer	119.7(27.5)	120.38	9.66(21.72)	63.85	46.03(26)	103.56	23.48(26.2)	92.22	315.8(22.4)	80.29
Colon and rectum cancer	41.69(9.57)	43.45	6.11(13.74)	43.92	22.07(12.5)	49.07	9.81(10.96)	40.55	139.6(9.88)	36.01
Stomach cancer	71.36(16.4)	71.88	6(13.49)	41.04	9.12(5.15)	20.73	2.4(2.68)	9.99	145.2(10.3)	36.89
Breast cancer	0.8(0.18)	0.77	0.02(0.04)	0.19	0.24(0.14)	0.56	0.16(0.18)	0.64	3.15(0.22)	0.8
Other malignant neoplasms	12.32(2.83)	14.64	1.13(2.54)	11.33	5.48(3.1)	15.64	2.78(3.1)	14.05	75.31(5.33)	19.44
Liver cancer	41.46(9.51)	41.49	3.89(8.75)	27.39	6.14(3.47)	14.08	3.97(4.43)	16.23	90.49(6.41)	22.53
Leukemia	13.65(3.13)	19.08	1.23(2.77)	12.18	6.18(3.49)	16.61	3.97(4.43)	18.15	66.71(4.72)	17.54
Esophageal cancer	46.22(10.6)	45.85	2.17(4.88)	15.49	6.5(3.67)	15.03	3.91(4.37)	15.73	88.22(6.24)	22.14
Pancreatic cancer	17.61(4.04)	17.64	3.33(7.49)	23.83	11.01(6.22)	24.97	6.26(6.99)	25.04	64.85(4.59)	16.47
Brain and central nervous system cancer	11.78(2.7)	14.3	0.51(1.15)	6.42	6.19(3.5)	18.35	3.28(3.66)	15.98	50.13(3.55)	12.76
Prostate cancer	10.03(2.3)	11.89	2.45(5.51)	13.94	17.37(9.81)	35.37	9.27(10.35)	35.87	86.45(6.12)	24.41
Non-Hodgkin lymphoma	8.79(2.02)	9.56	1.39(3.13)	10.28	4.93(2.78)	12.12	3.54(3.95)	14.94	42.18(2.99)	10.82
Lip and oral cavity cancer	4.55(1.04)	4.58	0.48(1.08)	3.83	3.41(1.93)	8.31	1.28(1.43)	5.26	37.59(2.66)	9.28
Bladder cancer	6.51(1.49)	7.35	1.07(2.41)	6.60	7.61(4.3)	16.1	2.82(3.15)	11.08	33.26(2.35)	9.02
Other leukemia	7.41(1.7)	9.99	0.32(0.72)	2.61	1.35(0.76)	3.40	0.93(1.04)	4.12	22.14(1.57)	5.80
Kidney cancer	4.63(1.06)	4.96	0.89(2)	6.49	5.6(3.16)	12.93	2.98(3.33)	12.25	27.36(1.94)	7.01
Gallbladder and biliary tract cancer	4.11(0.94)	4.26	1.56(3.51)	10.01	1.62(0.92)	3.57	0.43(0.48)	1.74	15.79(1.12)	4.10
Acute myeloid leukemia	1.68(0.39)	2.33	0.64(1.44)	5.99	2.44(1.38)	6.51	1.73(1.93)	7.75	17.28(1.22)	4.51
Larynx cancer	4.22(0.97)	4.15	0.2(0.45)	1.35	3.01(1.7)	7.01	0.95(1.06)	3.77	27.98(1.98)	6.93
Other pharynx cancer	1.22(0.28)	1.18	0.48(1.08)	3.53	2.93(1.66)	7.16	0.65(0.73)	2.65	25.03(1.77)	6.12
Acute lymphoid leukemia	3.4(0.78)	5.46	0.17(0.38)	2.75	0.71(0.4)	2.99	0.42(0.47)	2.64	16.07(1.14)	4.3

Multiple myeloma	2.04(0.47)	2.06	0.46(1.03)	3.10	2.82(1.59)	6.17	1.98(2.21)	7.85	13.77(0.97)	3.55
Nasopharynx cancer	6.93(1.59)	6.95	0.17(0.38)	1.38	0.56(0.32)	1.47	0.23(0.26)	1.04	16.84(1.19)	4.11
Malignant skin melanoma	0.8(0.18)	0.86	0.08(0.18)	0.73	2.63(1.49)	6.77	2.02(2.26)	8.79	9.74(0.69)	2.48
Other neoplasms	1.09(0.25)	1.22	0.56(1.26)	3.88	1.64(0.93)	3.50	1.06(1.18)	4.31	7.06(0.5)	1.95
Thyroid cancer	1.07(0.25)	1.16	0.13(0.29)	0.93	0.48(0.27)	1.17	0.31(0.35)	1.29	5.1(0.36)	1.29
Non-melanoma skin cancer	1.7(0.39)	1.89	0.13(0.29)	0.88	0.73(0.41)	1.63	1.03(1.15)	4.09	7.43(0.53)	2
Hodgkin lymphoma	0.57(0.13)	0.65	0.08(0.18)	0.65	0.5(0.28)	1.56	0.27(0.3)	1.37	7.26(0.51)	1.84
Chronic myeloid leukemia	0.27(0.06)	0.32	0.06(0.13)	0.58	0.39(0.22)	0.97	0.2(0.22)	0.90	6.36(0.45)	1.64
Chronic lymphoid leukemia	0.89(0.2)	0.98	0.04(0.09)	0.25	1.29(0.73)	2.75	0.7(0.78)	2.74	4.86(0.34)	1.3
Mesothelioma	0.51(0.12)	0.51	0.24(0.54)	1.71	1.62(0.92)	3.51	0.45(0.5)	1.78	4.71(0.33)	1.22
Testicular cancer	0.51(0.12)	0.67	0.05(0.11)	0.91	0.59(0.33)	2.34	0.27(0.3)	1.62	5.62(0.4)	1.42

DALYs, disability-adjusted life-years; ASDR, age-standardized DALY rate.

Table S3. The DALYs and age-standardized DALYs rate of all neoplasms in China, Japan, European Union, USA, and the world, 2019, women.

Neoplasm type	China		Japan		European Union		USA		Worldwide	
	DALYs $*10^5$ (%)	ASDR $/10^4$	DALYs $*10^5$ (%)	ASDR $/10^4$	DALYs $*10^5$ (%)	ASDR $/10^4$	DALYs $*10^5$ (%)	ASDR $/10^4$	DALYs $*10^5$ (%)	ASDR $/10^4$
Neoplasms	239.38(100)	240.97	29.69(100)	196.01	132.95(100)	272.36	76.98(100)	285.72	1101.2(100)	258.39
Tracheal, bronchus, and lung cancer	51.61(21.6)	49.22	3.81(12.83)	21.06	21.44(16.1)	43.21	18.38(23.9)	63.38	142.7(13.0)	32.76
Colon and rectum cancer	22.26(9.3)	21.73	4.45(14.99)	25.68	16.38(12.3)	29.57	7.8(10.13)	27.93	103.2(9.37)	23.79
Stomach cancer	26.89(11.2)	26.1	2.93(9.87)	17.3	5.4(4.06)	10.09	1.47(1.91)	5.45	77(6.99)	17.82
Breast cancer	28.77(12.0)	27.8	3.95(13.3)	33.81	25.62(19.3)	55.03	13.88(18.0)	53.64	203.1(18.4)	47.38
Other malignant neoplasms	8.39(3.5)	10.1	0.98(3.3)	9.02	5.15(3.87)	12.68	2.79(3.62)	12.74	59.11(5.37)	14.75
Liver cancer	11.8(4.93)	11.59	1.68(5.66)	8.58	2.66(2)	5.09	1.55(2.01)	5.69	34.8(3.16)	8.13
Leukemia	9.44(3.94)	13.6	0.78(2.63)	7.52	4.5(3.38)	10.71	2.71(3.52)	11.44	49.87(4.53)	12.72
Esophageal cancer	11.38(4.75)	10.85	0.38(1.28)	2.35	1.74(1.31)	3.23	0.81(1.05)	2.82	28.44(2.58)	6.53
Pancreatic cancer	10.45(4.37)	9.89	2.72(9.16)	14.96	9.43(7.09)	17.13	5.2(6.76)	17.8	50.64(4.6)	11.6
Brain and central nervous system cancer	8.75(3.66)	10.95	0.37(1.25)	4.46	4.19(3.15)	11.57	2.37(3.08)	10.96	36.47(3.31)	9.09
Cervical cancer	16.22(6.78)	15.75	0.97(3.27)	9.1	4.36(3.28)	10.62	2.25(2.92)	9.88	89.55(8.13)	21.06
Non-Hodgkin lymphoma	4.27(1.78)	4.69	0.94(3.17)	5.5	3.67(2.76)	7.31	2.54(3.3)	9.03	27.73(2.52)	6.61
Lip and oral cavity cancer	1.21(0.51)	1.19	0.29(0.98)	1.78	1.12(0.84)	2.3	0.53(0.69)	1.91	17.47(1.59)	4.08
Ovarian cancer	8.35(3.49)	8.05	1.27(4.28)	10.99	7.95(5.98)	16.76	4.27(5.55)	15.77	53.6(4.87)	12.47
Bladder cancer	1.65(0.69)	1.62	0.42(1.41)	1.81	2.26(1.7)	3.72	1.03(1.34)	3.33	10.66(0.97)	2.44
Other leukemia	5.16(2.16)	7.07	0.21(0.71)	1.58	0.99(0.74)	2.05	0.64(0.83)	2.51	17.24(1.57)	4.39
Kidney cancer	1.8(0.75)	2.01	0.41(1.38)	2.44	2.74(2.06)	5.34	1.37(1.78)	4.97	13.17(1.2)	3.11
Gallbladder and biliary tract cancer	3.53(1.47)	3.36	1.39(4.68)	6.77	2.2(1.65)	3.9	0.59(0.77)	2.06	20.42(1.85)	4.68

Acute myeloid leukemia	1.21(0.51)	1.74	0.38(1.28)	3.53	1.96(1.47)	4.82	1.29(1.68)	5.44	13.32(1.21)	3.34
Larynx cancer	0.76(0.32)	0.73	0.03(0.1)	0.18	0.35(0.26)	0.75	0.23(0.3)	0.83	4.64(0.42)	1.07
Other pharynx cancer	0.24(0.1)	0.23	0.06(0.2)	0.43	0.49(0.37)	1.08	0.17(0.22)	0.62	7.31(0.66)	1.7
Acute lymphoid leukemia	2.35(0.98)	4.01	0.13(0.44)	1.96	0.51(0.38)	2.03	0.31(0.4)	1.9	10.54(0.96)	2.89
Multiple myeloma	1.44(0.6)	1.38	0.41(1.38)	2.23	2.36(1.78)	4.17	1.53(1.99)	5.13	11.21(1.02)	2.57
Nasopharynx cancer	2.19(0.91)	2.2	0.03(0.1)	0.27	0.18(0.14)	0.44	0.09(0.12)	0.38	6.51(0.59)	1.54
Uterine cancer	3.64(1.52)	3.49	0.65(2.19)	5.09	3.69(2.78)	7.05	2.5(3.25)	8.79	23.29(2.11)	5.35
Malignant skin melanoma	0.7(0.29)	0.73	0.08(0.27)	0.65	2.02(1.52)	4.84	1.07(1.39)	4.46	7.34(0.67)	1.73
Other neoplasms	0.71(0.3)	0.76	0.32(1.08)	1.85	1.25(0.94)	1.99	0.73(0.95)	2.43	5.12(0.46)	1.19
Thyroid cancer	0.8(0.33)	0.81	0.22(0.74)	1.24	0.59(0.44)	1.19	0.32(0.42)	1.19	7.22(0.66)	1.69
Non-melanoma skin cancer	1.54(0.64)	1.52	0.07(0.24)	0.32	0.43(0.32)	0.68	0.46(0.6)	1.51	4.41(0.4)	1.02
Hodgkin lymphoma	0.29(0.12)	0.34	0.04(0.13)	0.29	0.38(0.29)	1.16	0.22(0.29)	1.1	4.2(0.38)	1.05
Chronic myeloid leukemia	0.14(0.06)	0.17	0.04(0.13)	0.33	0.29(0.22)	0.6	0.13(0.17)	0.52	4.14(0.38)	1.03
Chronic lymphoid leukemia	0.58(0.24)	0.6	0.02(0.07)	0.12	0.76(0.57)	1.21	0.35(0.45)	1.07	4.63(0.42)	1.06
Mesothelioma	0.28(0.12)	0.28	0.05(0.17)	0.34	0.39(0.29)	0.76	0.13(0.17)	0.48	1.97(0.18)	0.46

DALYs, disability-adjusted life-years; ASDR, age-standardized DALY rate.

Table S4. The estimated annual percentage change with 95% confidence interval in age-standardized DALYs rate of all neoplasms in China, Japan, European Union, USA, and the world, from 1990 to 2019, both sexes.

**Neoplasm type**	**China**	**Japan**	**European Union**	**USA**	**World**
Neoplasms	-1.26 (-1.37, -1.14)	-1.22 (-1.3, -1.15)	-0.96 (-1, -0.92)	-1.13 (-1.2, -1.07)	-0.9 (-0.94, -0.85)
Tracheal, bronchus, and lung cancer	0.42 (0.25, 0.59)	-0.9 (-1.01, -0.79)	-0.81 (-0.87, -0.75)	-1.83 (-1.94, -1.72)	-0.68 (-0.72, -0.63)
Colon and rectum cancer	1.24 (1, 1.49)	-0.82 (-0.88, -0.75)	-0.88 (-1, -0.76)	-1.1 (-1.21, -0.99)	-0.21 (-0.26, -0.15)
Stomach cancer	-1.98 (-2.4, -1.57)	-3.36 (-3.42, -3.31)	-2.76 (-2.83, -2.69)	-1.9 (-2.03, -1.77)	-2.11 (-2.28, -1.94)
Breast cancer	-0.13 (-0.19, -0.06)	0.42 (0.24, 0.6)	-1.48 (-1.54, -1.42)	-1.85 (-1.96, -1.74)	-0.5 (-0.56, -0.45)
Other malignant neoplasms	-1.36 (-1.46, -1.26)	-0.67 (-0.73, -0.62)	-1.33 (-1.38, -1.27)	-0.37 (-0.4, -0.33)	-0.43 (-0.48, -0.39)
Liver cancer	-5.31 (-6.17, -4.44)	-2.71 (-3.25, -2.15)	0.18 (0.11, 0.26)	2.59 (2.41, 2.78)	-2.78 (-3.24, -2.32)
Leukemia	-2.79 (-2.97, -2.61)	-1.95 (-2.03, -1.87)	-1.36 (-1.39, -1.32)	-1.27 (-1.33, -1.21)	-1.74 (-1.8, -1.69)
Esophageal cancer	-2.27 (-2.78, -1.75)	-1.08 (-1.26, -0.91)	-0.84 (-0.93, -0.74)	-0.16 (-0.27, -0.05)	-1.41 (-1.72, -1.11)
Pancreatic cancer	2.02 (1.83, 2.21)	0.17 (0.07, 0.27)	0.41 (0.35, 0.47)	0.35 (0.32, 0.38)	0.67 (0.62, 0.71)
Brain and central nervous system cancer	-1.13 (-1.28, -0.99)	1.02 (0.86, 1.19)	-0.44 (-0.49, -0.4)	-0.4 (-0.45, -0.34)	-0.44 (-0.49, -0.39)
Prostate cancer	-0.17 (-0.23, -0.11)	0.01 (-0.08, 0.1)	-0.46 (-0.59, -0.33)	-1.32 (-1.47, -1.17)	-0.49 (-0.56, -0.41)
Cervical cancer	0.22 (-0.03, 0.47)	-0.3 (-0.42, -0.18)	-1.95 (-1.99, -1.91)	-0.92 (-1.09, -0.76)	-0.94 (-0.99, -0.89)
Non-Hodgkin lymphoma	0.74 (0.51, 0.97)	-0.87 (-1, -0.75)	-1.11 (-1.28, -0.94)	-2.09 (-2.32, -1.85)	-0.28 (-0.35, -0.21)
Lip and oral cavity cancer	1.24 (0.96, 1.52)	-0.01 (-0.36, 0.33)	-1.26 (-1.35, -1.17)	-1.36 (-1.54, -1.19)	-0.12 (-0.15, -0.08)
Ovarian cancer	1.23 (1.13, 1.33)	-0.6 (-0.66, -0.54)	-1.25 (-1.32, -1.19)	-1.29 (-1.4, -1.18)	-0.11 (-0.15, -0.07)
Bladder cancer	-0.58 (-0.64, -0.52)	-0.64 (-0.7, -0.58)	-0.92 (-0.98, -0.86)	-0.14 (-0.19, -0.09)	-0.83 (-0.86, -0.79)
Other leukemia	-4.39 (-4.74, -4.04)	-2.67 (-2.82, -2.52)	-2.39 (-2.59, -2.19)	-1.69 (-1.77, -1.6)	-3.09 (-3.23, -2.95)
Kidney cancer	2.28 (1.83, 2.73)	-0.02 (-0.15, 0.11)	0.11 (0, 0.23)	-0.65 (-0.73, -0.57)	0.12 (0.04, 0.21)
Gallbladder and biliary tract cancer	0.93 (0.49, 1.37)	-2.17 (-2.23, -2.12)	-2.11 (-2.17, -2.04)	-1.29 (-1.36, -1.22)	-0.67 (-0.74, -0.6)
Acute myeloid leukemia	0.24 (0.14, 0.34)	-0.74 (-0.83, -0.65)	0.2 (0.12, 0.28)	-0.05 (-0.15, 0.06)	-0.05 (-0.08, -0.03)
Larynx cancer	-1.21 (-1.3, -1.12)	-2.44 (-2.51, -2.38)	-2.66 (-2.74, -2.59)	-2.02 (-2.14, -1.89)	-1.65 (-1.74, -1.57)
Other pharynx cancer	-1.21 (-1.39, -1.04)	1.77 (1.36, 2.19)	-0.31 (-0.45, -0.18)	-0.43 (-0.61, -0.24)	0.1 (0.05, 0.14)

Acute lymphoid leukemia	0.59 (0.19, 1)	-2.03 (-2.11, -1.95)	-2.03 (-2.1, -1.96)	-1.42 (-1.5, -1.33)	-0.81 (-0.89, -0.74)
Multiple myeloma	0.17 (0.1, 0.25)	-0.66 (-0.83, -0.5)	0.14 (-0.05, 0.32)	-0.6 (-0.72, -0.48)	-0.16 (-0.23, -0.08)
Nasopharynx cancer	-2.71 (-2.87, -2.56)	-0.37 (-0.74, -0.01)	-1.78 (-1.85, -1.71)	-1.65 (-1.8, -1.51)	-1.58 (-1.68, -1.48)
Uterine cancer	-2.14 (-2.73, -1.54)	0.69 (0.56, 0.83)	-0.1 (-0.17, -0.02)	0.62 (0.51, 0.74)	-0.87 (-0.96, -0.79)
Malignant skin melanoma	-0.56 (-0.69, -0.43)	0.05 (-0.12, 0.21)	0.39 (0.29, 0.49)	-0.59 (-0.65, -0.53)	-0.49 (-0.57, -0.41)
Other neoplasms	0.74 (0.67, 0.81)	0.55 (0.51, 0.59)	0.6 (0.49, 0.71)	0.15 (0.11, 0.2)	0.24 (0.22, 0.27)
Thyroid cancer	-0.2 (-0.31, -0.1)	-0.91 (-1.01, -0.81)	-1.6 (-1.66, -1.53)	0.4 (0.31, 0.49)	-0.14 (-0.18, -0.09)
Non-melanoma skin cancer	1.09 (0.81, 1.37)	0.44 (0.37, 0.52)	-0.7 (-0.81, -0.59)	-0.04 (-0.5, 0.43)	0.2 (0.1, 0.3)
Hodgkin lymphoma	-5.07 (-5.4, -4.75)	0.15 (0.01, 0.28)	-2.83 (-3.03, -2.64)	-2.53 (-2.66, -2.41)	-1.89 (-1.99, -1.78)
Chronic myeloid leukemia	-3.14 (-3.32, -2.95)	-6.19 (-6.57, -5.81)	-4.79 (-5.11, -4.47)	-5.27 (-5.75, -4.78)	-2.69 (-2.81, -2.58)
Chronic lymphoid leukemia	2.25 (2, 2.51)	-0.96 (-1.05, -0.87)	-0.26 (-0.51, 0)	-1.54 (-1.73, -1.35)	-0.34 (-0.4, -0.27)
Mesothelioma	0.95 (0.61, 1.3)	1.27 (1, 1.54)	0.01 (-0.05, 0.07)	-1.36 (-1.54, -1.18)	-0.47 (-0.52, -0.42)
Testicular cancer	-0.29 (-0.51, -0.07)	-1.88 (-2.12, -1.65)	-0.86 (-1.01, -0.72)	-0.62 (-0.71, -0.53)	-0.1 (-0.18, -0.02)

DALYs, disability-adjusted life-years

Table S5. The estimated annual percentage change with 95% confidence interval in age-standardized DALYs rate of all neoplasms in China, Japan, European Union, USA, and the world, from 1990 to 2019, men.

Neoplasm type	China	Japan	European Union	USA	World
Neoplasms	-0.96 (-1.05, -0.86)	-1.44 (-1.54, -1.35)	-1.18 (-1.23, -1.13)	-1.33 (-1.42, -1.24)	-0.94 (-0.98, -0.89)
Tracheal, bronchus, and lung cancer	0.6 (0.43, 0.77)	-1.1 (-1.24, -0.96)	-1.68 (-1.74, -1.63)	-2.47 (-2.58, -2.37)	-0.98 (-1.02, -0.94)
Colon and rectum cancer	2.12 (1.81, 2.43)	-0.77 (-0.85, -0.7)	-0.74 (-0.87, -0.61)	-1.15 (-1.28, -1.02)	0.13 (0.06, 0.2)
Stomach cancer	-1.47 (-1.89, -1.05)	-3.27 (-3.32, -3.21)	-2.88 (-2.95, -2.81)	-2.14 (-2.29, -1.99)	-1.93 (-2.12, -1.73)
Breast cancer	6.59 (5.51, 7.67)	-0.59 (-0.84, -0.34)	-0.22 (-0.56, 0.12)	-1.23 (-1.51, -0.96)	0.14 (-0.01, 0.29)
Other malignant neoplasms	-0.68 (-0.76, -0.61)	-0.89 (-0.96, -0.82)	-1.71 (-1.8, -1.61)	-0.57 (-0.63, -0.52)	-0.39 (-0.45, -0.33)
Liver cancer	-5.05 (-5.94, -4.15)	-2.94 (-3.45, -2.42)	0.18 (0.1, 0.26)	2.73 (2.55, 2.92)	-2.79 (-3.28, -2.3)
Leukemia	-2.33 (-2.43, -2.24)	-1.83 (-1.91, -1.75)	-1.29 (-1.32, -1.26)	-1.29 (-1.35, -1.23)	-1.49 (-1.52, -1.46)
Esophageal cancer	-1.56 (-2.03, -1.1)	-1.26 (-1.46, -1.07)	-0.99 (-1.09, -0.9)	-0.11 (-0.23, 0.01)	-1.08 (-1.37, -0.79)
Pancreatic cancer	2.39 (2.17, 2.61)	-0.05 (-0.15, 0.05)	0.22 (0.15, 0.29)	0.31 (0.26, 0.35)	0.68 (0.62, 0.74)
Brain and central nervous system cancer	-0.94 (-1.04, -0.84)	1.11 (0.94, 1.29)	-0.3 (-0.35, -0.25)	-0.39 (-0.45, -0.32)	-0.39 (-0.42, -0.35)
Prostate cancer	-0.24 (-0.27, -0.2)	-0.33 (-0.44, -0.23)	-0.91 (-1.05, -0.77)	-1.7 (-1.86, -1.54)	-0.71 (-0.78, -0.63)
Non-Hodgkin lymphoma	1.66 (1.37, 1.95)	-0.9 (-1.01, -0.8)	-1.13 (-1.27, -0.98)	-2.1 (-2.34, -1.87)	-0.12 (-0.19, -0.05)
Lip and oral cavity cancer	2.41 (2.02, 2.81)	-0.27 (-0.61, 0.08)	-1.66 (-1.74, -1.57)	-1.42 (-1.62, -1.22)	-0.24 (-0.28, -0.2)
Bladder cancer	-0.07 (-0.12, -0.02)	-0.81 (-0.88, -0.75)	-1.19 (-1.25, -1.13)	-0.24 (-0.28, -0.2)	-0.84 (-0.87, -0.81)
Other leukemia	-3.88 (-4.18, -3.58)	-2.49 (-2.66, -2.32)	-2.19 (-2.39, -2)	-1.63 (-1.71, -1.55)	-2.72 (-2.83, -2.61)
Kidney cancer	3.33 (2.82, 3.85)	-0.06 (-0.19, 0.06)	0.15 (0.03, 0.26)	-0.55 (-0.64, -0.46)	0.4 (0.3, 0.5)
Gallbladder and biliary tract cancer	1.54 (1.1, 1.99)	-1.57 (-1.64, -1.51)	-1.37 (-1.42, -1.32)	-1.21 (-1.3, -1.13)	-0.21 (-0.32, -0.11)
Acute myeloid leukemia	0.33 (0.21, 0.44)	-0.56 (-0.66, -0.46)	0.29 (0.2, 0.37)	-0.01 (-0.1, 0.07)	0.11 (0.07, 0.14)
Larynx cancer	-0.94 (-1.04, -0.84)	-2.69 (-2.77, -2.61)	-2.92 (-3, -2.85)	-2.16 (-2.29, -2.03)	-1.74 (-1.83, -1.65)
Other pharynx cancer	-0.91 (-1.12, -0.69)	1.8 (1.36, 2.24)	-0.54 (-0.66, -0.41)	-0.33 (-0.53, -0.14)	0.15 (0.1, 0.19)
Acute lymphoid leukemia	0.84 (0.33, 1.35)	-1.99 (-2.07, -1.91)	-2.03 (-2.11, -1.95)	-1.53 (-1.61, -1.44)	-0.65 (-0.74, -0.55)
Multiple myeloma	0.87 (0.8, 0.94)	-0.59 (-0.78, -0.4)	0.19 (0, 0.37)	-0.59 (-0.71, -0.48)	0.01 (-0.05, 0.08)

Nasopharynx cancer	-2.1 (-2.22, -1.98)	-0.3 (-0.68, 0.09)	-1.94 (-2.01, -1.87)	-1.62 (-1.77, -1.47)	-1.21 (-1.27, -1.14)
Malignant skin melanoma	-0.55 (-0.67, -0.43)	-0.13 (-0.28, 0.03)	0.54 (0.42, 0.66)	-0.54 (-0.6, -0.47)	-0.33 (-0.42, -0.24)
Other neoplasms	1.15 (1.05, 1.25)	0.46 (0.42, 0.49)	0.31 (0.21, 0.41)	0.01 (-0.04, 0.06)	0.19 (0.17, 0.22)
Thyroid cancer	1.84 (1.56, 2.13)	-0.47 (-0.63, -0.31)	-0.94 (-1.01, -0.88)	0.46 (0.36, 0.56)	0.61 (0.53, 0.68)
Non-melanoma skin cancer	0.99 (0.73, 1.25)	0.59 (0.48, 0.69)	-0.53 (-0.6, -0.45)	-0.25 (-0.69, 0.18)	0.2 (0.09, 0.3)
Hodgkin lymphoma	-4.69 (-4.97, -4.4)	0.1 (-0.06, 0.26)	-3.24 (-3.47, -3.02)	-2.93 (-3.11, -2.75)	-1.95 (-2.04, -1.86)
Chronic myeloid leukemia	-2.45 (-2.56, -2.33)	-6.19 (-6.55, -5.83)	-4.68 (-5, -4.36)	-5.19 (-5.67, -4.7)	-2.45 (-2.55, -2.35)
Chronic lymphoid leukemia	2.72 (2.43, 3.01)	-0.84 (-0.98, -0.7)	-0.3 (-0.56, -0.03)	-1.5 (-1.68, -1.32)	-0.49 (-0.59, -0.39)
Mesothelioma	2.42 (1.92, 2.91)	1.71 (1.38, 2.04)	0.04 (-0.03, 0.11)	-1.73 (-1.94, -1.52)	-0.23 (-0.31, -0.15)
Testicular cancer	-0.25 (-0.47, -0.04)	-1.91 (-2.14, -1.67)	-0.9 (-1.04, -0.75)	-0.64 (-0.73, -0.54)	-0.11 (-0.18, -0.03)

DALYs, disability-adjusted life-years

Table S6. The estimated annual percentage change with 95% confidence interval in age-standardized DALYs rate of all neoplasms in China, Japan, European Union, USA, and the world, from 1990 to 2019, women.

Neoplasm type	China	Japan	European Union	USA	World
Neoplasms	-1.68 (-1.82, -1.54)	-1 (-1.05, -0.96)	-0.77 (-0.8, -0.74)	-1.01 (-1.07, -0.95)	-0.86 (-0.92, -0.81)
Tracheal, bronchus, and lung cancer	0.11 (-0.07, 0.3)	-0.83 (-0.9, -0.77)	1.47 (1.3, 1.65)	-1.01 (-1.19, -0.83)	0.07 (-0.02, 0.16)
Colon and rectum cancer	-0.03 (-0.19, 0.13)	-1.01 (-1.07, -0.96)	-1.17 (-1.29, -1.06)	-1.15 (-1.24, -1.05)	-0.65 (-0.71, -0.6)
Stomach cancer	-3.02 (-3.41, -2.63)	-3.75 (-3.85, -3.66)	-2.76 (-2.84, -2.67)	-1.68 (-1.78, -1.57)	-2.46 (-2.59, -2.33)
Breast cancer	-0.35 (-0.43, -0.26)	0.5 (0.31, 0.68)	-1.41 (-1.47, -1.35)	-1.77 (-1.88, -1.66)	-0.51 (-0.57, -0.45)
Other malignant neoplasms	-2.18 (-2.39, -1.98)	-0.43 (-0.47, -0.39)	-0.89 (-0.92, -0.86)	-0.16 (-0.19, -0.14)	-0.5 (-0.55, -0.45)
Liver cancer	-5.87 (-6.62, -5.1)	-2.03 (-2.68, -1.37)	-0.07 (-0.19, 0.05)	2.06 (1.89, 2.24)	-2.68 (-3.05, -2.3)
Leukemia	-3.37 (-3.67, -3.08)	-2.14 (-2.22, -2.06)	-1.48 (-1.52, -1.43)	-1.31 (-1.37, -1.24)	-2.07 (-2.17, -1.98)
Esophageal cancer	-4.1 (-4.77, -3.43)	-0.75 (-0.79, -0.71)	-0.54 (-0.63, -0.45)	-0.85 (-0.93, -0.77)	-2.31 (-2.65, -1.96)
Pancreatic cancer	1.45 (1.29, 1.61)	0.36 (0.26, 0.47)	0.61 (0.56, 0.66)	0.36 (0.33, 0.4)	0.66 (0.63, 0.68)
Brain and central nervous system cancer	-1.37 (-1.58, -1.17)	0.88 (0.73, 1.03)	-0.67 (-0.72, -0.62)	-0.44 (-0.5, -0.39)	-0.51 (-0.57, -0.44)
Cervical cancer	0.16 (-0.09, 0.41)	-0.13 (-0.25, -0.01)	-1.88 (-1.92, -1.84)	-0.86 (-1.02, -0.7)	-0.95 (-1, -0.89)
Non-Hodgkin lymphoma	-0.75 (-1.03, -0.46)	-0.85 (-1.01, -0.69)	-1.08 (-1.29, -0.87)	-2.08 (-2.33, -1.83)	-0.52 (-0.63, -0.42)
Lip and oral cavity cancer	-1.57 (-1.66, -1.48)	0.35 (0.02, 0.68)	0.08 (-0.04, 0.2)	-1.4 (-1.53, -1.27)	0.14 (0.06, 0.22)
Ovarian cancer	1.15 (1.04, 1.25)	-0.5 (-0.56, -0.44)	-1.15 (-1.22, -1.08)	-1.18 (-1.29, -1.07)	-0.08 (-0.12, -0.04)
Bladder cancer	-2.2 (-2.29, -2.12)	-0.71 (-0.75, -0.67)	-0.71 (-0.77, -0.66)	-0.34 (-0.41, -0.27)	-1.04 (-1.08, -0.99)
Other leukemia	-5.04 (-5.46, -4.6)	-2.95 (-3.07, -2.84)	-2.68 (-2.88, -2.48)	-1.83 (-1.91, -1.74)	-3.52 (-3.7, -3.34)
Kidney cancer	0.55 (0.19, 0.9)	-0.15 (-0.3, 0.01)	-0.13 (-0.25, -0.01)	-1.02 (-1.11, -0.93)	-0.41 (-0.46, -0.36)
Gallbladder and biliary tract cancer	0.3 (-0.14, 0.75)	-2.84 (-2.94, -2.75)	-2.54 (-2.63, -2.45)	-1.32 (-1.38, -1.26)	-0.98 (-1.03, -0.93)
Acute myeloid leukemia	0.14 (-0.01, 0.29)	-1.04 (-1.13, -0.95)	0.07 (-0.01, 0.15)	-0.14 (-0.27, -0.02)	-0.25 (-0.28, -0.22)
Larynx cancer	-2.23 (-2.41, -2.06)	-2.19 (-2.3, -2.08)	-1 (-1.03, -0.96)	-1.93 (-2.05, -1.8)	-1.16 (-1.23, -1.09)
Other pharynx cancer	-2.38 (-2.47, -2.3)	0.86 (0.67, 1.05)	0.92 (0.77, 1.08)	-1.05 (-1.22, -0.87)	-0.06 (-0.16, 0.04)
Acute lymphoid leukemia	0.25 (-0.07, 0.57)	-2.09 (-2.18, -2)	-2.04 (-2.11, -1.97)	-1.26 (-1.35, -1.18)	-1.06 (-1.12, -1)

Multiple myeloma	-0.63 (-0.73, -0.54)	-0.83 (-0.97, -0.69)	0.01 (-0.18, 0.19)	-0.69 (-0.83, -0.55)	-0.38 (-0.46, -0.29)
Nasopharynx cancer	-4.12 (-4.37, -3.87)	-0.97 (-1.24, -0.7)	-1.44 (-1.52, -1.35)	-1.86 (-2, -1.71)	-2.4 (-2.58, -2.22)
Uterine cancer	-2.21 (-2.81, -1.6)	0.92 (0.78, 1.05)	0.07 (-0.01, 0.16)	0.82 (0.71, 0.93)	-0.84 (-0.93, -0.75)
Malignant skin melanoma	-0.55 (-0.7, -0.41)	0.2 (0.02, 0.38)	0.19 (0.11, 0.28)	-0.74 (-0.8, -0.68)	-0.68 (-0.75, -0.61)
Other neoplasms	0.17 (0.09, 0.25)	0.52 (0.47, 0.57)	0.76 (0.64, 0.89)	0.11 (0.06, 0.16)	0.22 (0.19, 0.25)
Thyroid cancer	-2 (-2.11, -1.89)	-1.13 (-1.21, -1.05)	-2.02 (-2.12, -1.93)	0.36 (0.24, 0.49)	-0.57 (-0.62, -0.52)
Non-melanoma skin cancer	1.16 (0.86, 1.46)	-0.11 (-0.19, -0.03)	-1.16 (-1.36, -0.96)	0.26 (-0.28, 0.81)	0.15 (0.04, 0.26)
Hodgkin lymphoma	-5.67 (-6.08, -5.26)	0 (-0.08, 0.09)	-2.24 (-2.38, -2.09)	-2 (-2.06, -1.93)	-1.77 (-1.9, -1.64)
Chronic myeloid leukemia	-4.15 (-4.49, -3.8)	-6.13 (-6.53, -5.74)	-4.97 (-5.28, -4.65)	-5.43 (-5.92, -4.94)	-3.06 (-3.21, -2.91)
Chronic lymphoid leukemia	1.56 (1.27, 1.86)	-1.34 (-1.43, -1.25)	-0.41 (-0.64, -0.17)	-1.81 (-2.03, -1.6)	-0.19 (-0.26, -0.12)
Mesothelioma	-0.89 (-1.03, -0.75)	-0.68 (-0.75, -0.62)	-0.58 (-0.66, -0.5)	-0.53 (-0.59, -0.48)	-1.1 (-1.14, -1.06)

DALYs, disability-adjusted life-years

Table S7. The proportion (%) of cancer DALYs attributable to risk factors in the world, China, Japan, European Union, and USA, 1990, stratified by sex.

GBD risk factors	Worldwide			China			Japan			European Union			USA		
	Both	Men	Women	Both	Men	Women	Both	Men	Women	Both	Men	Women	Both	Men	Women
All risk factors	41.51	48.39	32.62	39.84	48.52	26.56	42.25	51.18	29.59	47.24	54.67	37.01	49.06	52.96	44.30
Smoking	24.82	36.58	9.65	23.92	36.63	4.49	28.15	39.76	11.79	30.63	41.38	16.15	34.43	41.22	26.45
Alcohol use	4.95	6.54	2.74	5.24	7.35	1.75	5.19	6.09	3.73	7.52	8.59	5.86	4.29	4.45	4.07
Unsafe sex	3.66	NA	8.65	1.86	NA	4.84	1.61	NA	4.06	2.22	NA	5.40	1.57	NA	3.47
Occupational carcinogens	2.94	4.49	1.11	1.50	1.70	1.18	2.55	3.82	0.98	5.41	8.64	1.41	5.58	9.18	1.84
High body-mass index	2.87	2.47	3.30	2.00	1.96	1.99	2.43	2.47	2.22	4.00	3.52	4.45	4.44	4.02	4.73
High fasting plasma glucose	2.19	2.19	2.21	1.49	1.44	1.62	2.14	2.50	1.70	2.98	3.06	2.90	4.55	4.54	4.54
Ambient particulate matter pollution	1.91	2.64	0.96	1.73	2.14	1.12	1.34	1.68	0.88	2.91	4.13	1.21	2.58	3.11	1.95
Household air pollution from solid fuels	1.71	2.13	1.15	3.59	3.93	3.08	0.02	0.03	0.02	0.29	0.38	0.14	0.01	0.01	0.01
Diet low in fruits	1.65	2.05	1.10	2.76	3.10	2.20	1.17	1.54	0.63	1.02	1.39	0.49	1.39	1.70	1.01
Diet low in whole grains	1.29	1.22	1.38	0.81	0.73	0.94	1.64	1.58	1.71	1.84	1.76	1.95	1.58	1.61	1.52
Secondhand smoke	1.27	1.22	1.34	1.42	1.08	1.99	0.95	0.82	1.15	1.56	1.81	1.17	2.10	2.41	1.69
Diet low in milk	1.14	1.08	1.22	0.99	0.88	1.16	2.03	1.92	2.16	1.30	1.23	1.38	1.03	1.04	1.01
Diet low in calcium	1.02	1.01	1.02	1.08	1.00	1.22	1.57	1.56	1.56	0.66	0.72	0.59	0.55	0.63	0.45
Diet high in sodium	1.01	1.15	0.81	1.72	1.85	1.50	1.86	1.99	1.66	0.50	0.60	0.38	0.16	0.19	0.13
Residential radon	0.77	1.04	0.42	0.61	0.70	0.48	0.24	0.30	0.16	1.19	1.68	0.51	1.25	1.50	0.95
Diet high in red meat	0.66	0.39	1.02	0.28	0.16	0.47	0.45	0.31	0.65	1.32	0.77	2.09	1.35	0.81	1.98
Drug use	0.65	0.68	0.60	1.52	1.43	1.67	1.27	1.49	0.81	0.35	0.42	0.25	0.35	0.35	0.34
Chewing tobacco	0.44	0.49	0.37	0.08	0.11	0.03	0.04	0.06	0.02	0.02	0.02	0.01	0.18	0.31	0.03
Low physical activity	0.43	0.28	0.61	0.19	0.13	0.28	0.62	0.48	0.83	0.72	0.46	1.05	0.59	0.30	0.90
Diet low in vegetables	0.35	0.42	0.25	0.71	0.80	0.55	0.11	0.16	0.04	0.13	0.18	0.06	0.10	0.14	0.04
Diet high in processed meat	0.31	0.28	0.34	0.04	0.03	0.05	0.60	0.55	0.66	0.61	0.55	0.67	0.66	0.66	0.65
Diet low in vegetables	0.16	0.20	0.12	0.03	0.04	0.02	0.06	0.08	0.02	0.13	0.19	0.06	0.15	0.23	0.05

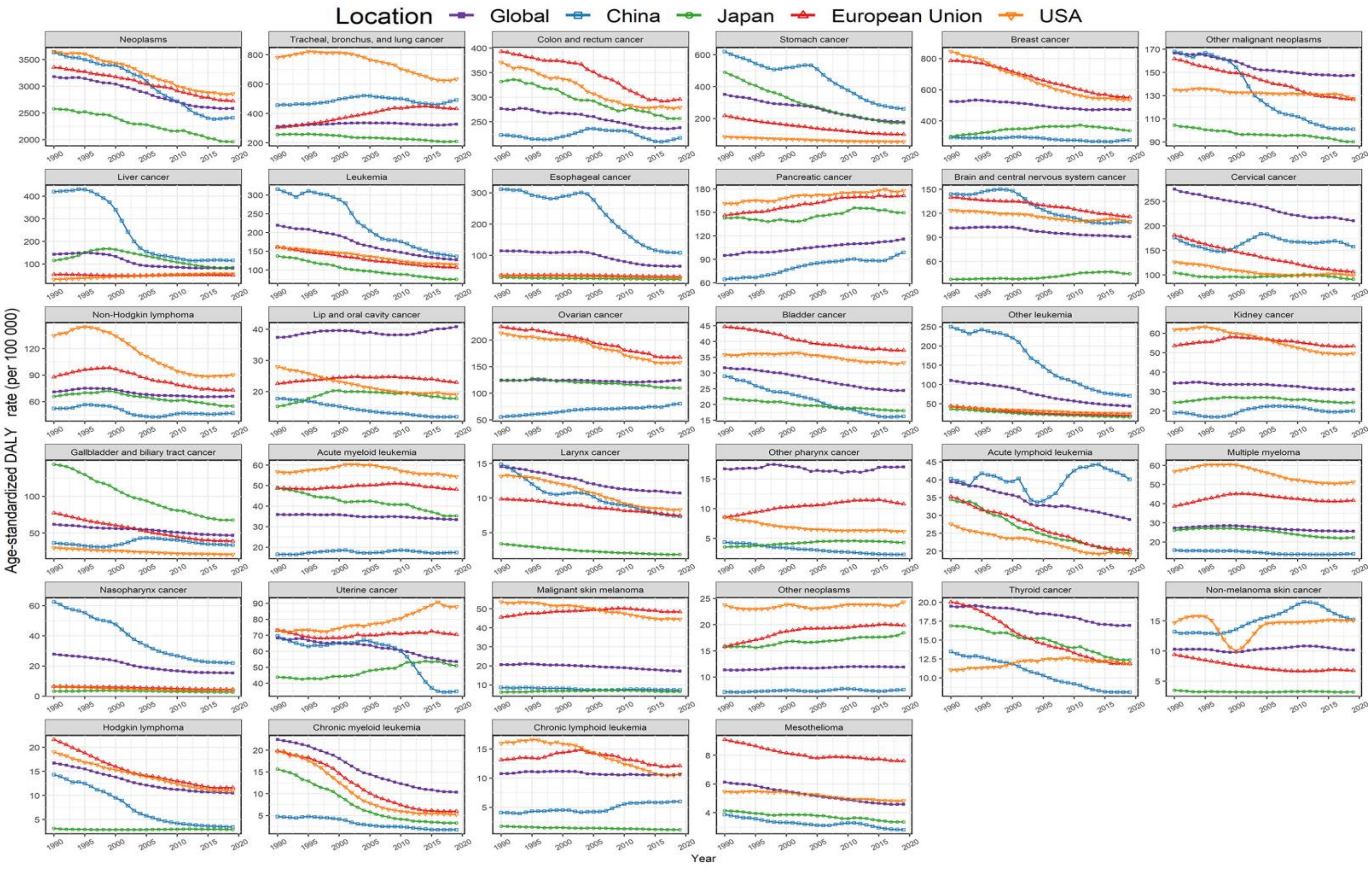

Figure S1. Trends for the ASDR of 36 neoplasm types in the world, China, Japan, European Union, and USA from 1990 to 2019, men. ASDR, age-standardized disability-adjusted life-year rate.

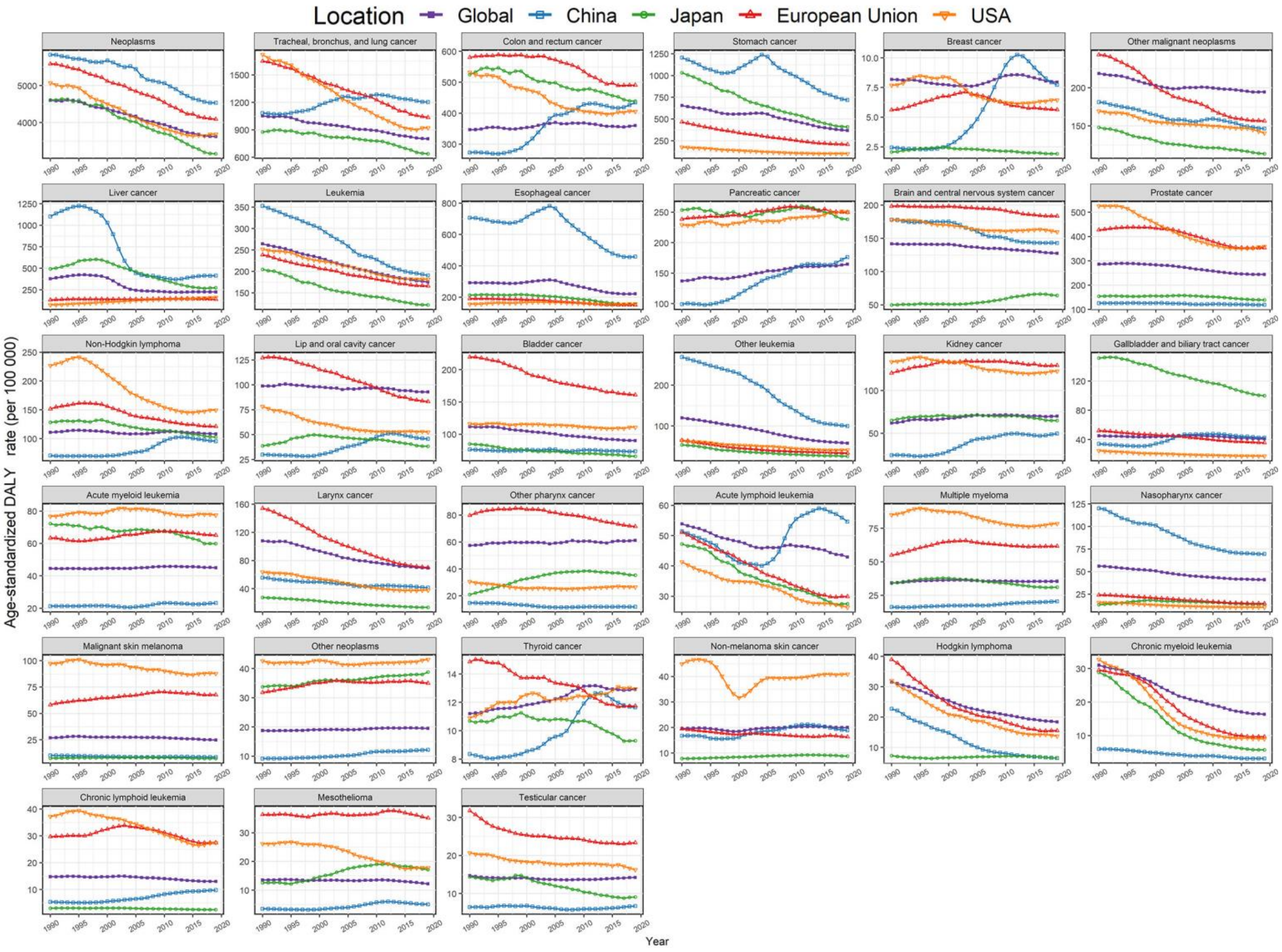

Figure S2. Trends for the ASDR of 36 neoplasm types in the world, China, Japan, European Union, and USA from 1990 to 2019, women. ASDR, age-standardized disability-adjusted life-year rate

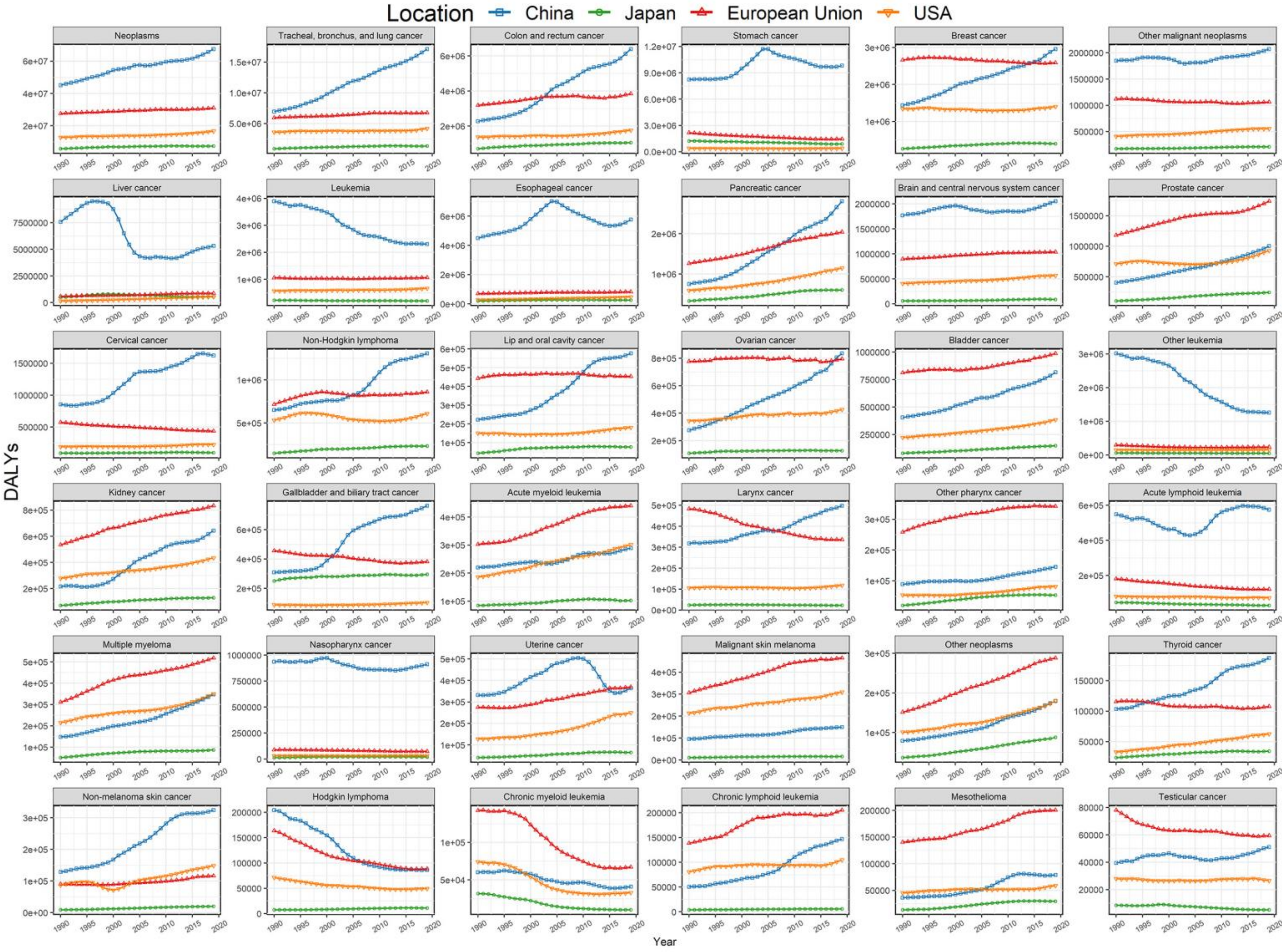

Figure S3. Trends for the absolute DALYs of 36 neoplasm types in China, Japan, European Union, and USA from 1990 to 2019, both. DALYs, disability-adjusted life-years.
